# Metaball skinning of synthetic astroglial morphologies into realistic mesh models for *in silico* simulations and visual analytics

**DOI:** 10.1093/bioinformatics/btab280

**Published:** 2021-07-12

**Authors:** Marwan Abdellah, Alessandro Foni, Eleftherios Zisis, Nadir Román Guerrero, Samuel Lapere, Jay S Coggan, Daniel Keller, Henry Markram, Felix Schürmann

**Affiliations:** Blue Brain Project (BBP), École polytechnique fédérale de Lausanne (EPFL), Geneva 1202, Switzerland; Blue Brain Project (BBP), École polytechnique fédérale de Lausanne (EPFL), Geneva 1202, Switzerland; Blue Brain Project (BBP), École polytechnique fédérale de Lausanne (EPFL), Geneva 1202, Switzerland; Blue Brain Project (BBP), École polytechnique fédérale de Lausanne (EPFL), Geneva 1202, Switzerland; Blue Brain Project (BBP), École polytechnique fédérale de Lausanne (EPFL), Geneva 1202, Switzerland; Blue Brain Project (BBP), École polytechnique fédérale de Lausanne (EPFL), Geneva 1202, Switzerland; Blue Brain Project (BBP), École polytechnique fédérale de Lausanne (EPFL), Geneva 1202, Switzerland; Blue Brain Project (BBP), École polytechnique fédérale de Lausanne (EPFL), Geneva 1202, Switzerland; Blue Brain Project (BBP), École polytechnique fédérale de Lausanne (EPFL), Geneva 1202, Switzerland

## Abstract

**Motivation:**

Astrocytes, the most abundant glial cells in the mammalian brain, have an instrumental role in developing neuronal circuits. They contribute to the physical structuring of the brain, modulating synaptic activity and maintaining the blood–brain barrier in addition to other significant aspects that impact brain function. Biophysically, detailed astrocytic models are key to unraveling their functional mechanisms via molecular simulations at microscopic scales. Detailed, and complete, biological reconstructions of astrocytic cells are sparse. Nonetheless, data-driven digital reconstruction of astroglial morphologies that are statistically identical to biological counterparts are becoming available. We use those synthetic morphologies to generate astrocytic meshes with realistic geometries, making it possible to perform these simulations.

**Results:**

We present an unconditionally robust method capable of reconstructing high fidelity polygonal meshes of astroglial cells from algorithmically-synthesized morphologies. Our method uses implicit surfaces, or metaballs, to skin the different structural components of astrocytes and then blend them in a seamless fashion. We also provide an end-to-end pipeline to produce optimized two- and three-dimensional meshes for visual analytics and simulations, respectively. The performance of our pipeline has been assessed with a group of 5000 astroglial morphologies and the geometric metrics of the resulting meshes are evaluated. The usability of the meshes is then demonstrated with different use cases.

**Availability and implementation:**

Our metaball skinning algorithm is implemented in Blender 2.82 relying on its Python API (Application Programming Interface). To make it accessible to computational biologists and neuroscientists, the implementation has been integrated into NeuroMorphoVis, an open source and domain specific package that is primarily designed for neuronal morphology visualization and meshing.

**Supplementary information:**

Supplementary data are available at *Bioinformatics* online.

## 1 Introduction

Most mammalian brains are comprised of two broad categories of cell types, neurons and glia, each of which contains a variety of constituent subtypes. Although neurons are by far the most widely studied and considered the primary substrates of behavior, glia are often just as numerous (Jäkel and Dimou, 2017; Sherwood *et al.*, 2006). Mammalian glia subtypes include astrocytes, oligodendrocytes and microglia (Bullock, 2004), with astrocytes being the most abundant of the three. A higher glia-to-neuron ratio in humans is thought to reflect higher energy needs, since astrocytes form an energy processing unit with neurons and local capillaries (Bélanger *et al.*, 2011; Herculano-Houzel, 2014). Much evidence points to a critical role for astrocytes in the normal formation of developing neuronal circuits as well as their function in maturity (Khakh and Sofroniew, 2015). Brain astrocytes are involved in modulating synaptic activity, integrating intracellular and extracellular signals and responding to or mediating pathological states, including numerous neurodegenerative disorders such as Parkinson’s and Alzheimer’s diseases (Rossi and Volterra, 2009). The dysfunction of astrocytes is also implicated in the pathogenic neuronal activity seen in ischemic stroke and epilepsy in the prefrontal cortex (Siracusa *et al.*, 2019). In short, there is clear evidence for glial contributions to almost every conceivable brain function or disorder. Almost none of the multiple aspects of neuro-glia signaling briefly described here, as well as many others not mentioned, can be understood at a macroscopic or whole-cell scale. On the contrary, almost all of the functions carried out by astrocytes depend on finer scale ultrastructural domains. For example, it is the endfeet of astrocytes that interface with the vasculature, forming part of the blood–brain barrier (Cabezas *et al.*, 2014; Mathiisen *et al.*, 2010) and specialized, thin, processes envelop synapses, forming the canonical tripartite synapse structure. Furthermore, local intracellular components must conform to corresponding spatial constraints, such as the locating of mitochondria or glycogen to support energy demands (Calì *et al.*, 2019) or calcium signaling components (Bazargani and Attwell, 2016). Moreover, molecular and morphological differences among astrocytes can be region specific (Lanjakornsiripan *et al.*, 2018). Given their elaborate anatomical features and extensive domains, it is difficult to imagine how one can proceed to unravel the mechanisms of astrocytic functions without detailed, ultrastructurally accurate models that are essential to complement our understanding of the various roles and functional aspects of astrocytes in our brains (Bushong *et al.*, 2002).

Not surprisingly, the literature is comparatively sparse; few biologically detailed reconstructions of astrocytic cells exist. Therefore, it was essential to algorithmically synthesize digital reconstructions of the neuro-glia-vascular (NGV) ensemble including astroglial morphologies that are validated later against realistic biological counterparts to ensure their fidelity (Zisis *et al.*, 2021). Starting from this point, we can synthesize astrocytic models that can be used to study their functional aspects, which will help us to advance our limited understanding. This step is subject to the presence of a convenient method for building detailed and realistic geometric models from those synthetic morphologies, making it possible to perform accurate stochastic reaction–diffusion simulations. We address this concern and present a robust method to reconstruct high fidelity and adaptively-tessellated mesh models of astroglial cells from their morphological descriptions.

### 1.1 Relevant work

Accurate meshing of astroglial morphologies is relatively challenging compared to the other constituents of the NGV ensemble (Coggan *et al.*, 2018). Although their processes have branching topologies and hierarchical representations similar to neurons and vascular networks, astrocytes have complex sponge-like spatial appearance with extremely fine and convoluted fibers that interface with neurons and synapses, in addition to their perivascular endfeet that are wrapped around cerebrovasculature (blood vessels that feed the diverse structures of the brain). Unlike neuronal and vascular morphologies that are represented by acyclic and cyclic graphs respectively, astrocytic endfeet cannot be logically represented by graphs. Therefore, endfeet data of astroglial morphologies are ordinarily missing. In fact, there is no standard file format that would make it easy to have complete astroglial reconstructions available online. This is contrary to neurons which have a plethora of open-access SWC morphologies from the NeuroMorpho.Org database (Ascoli *et al.*, 2007) and even vasculature, where neurobiologists can find multiple arterial arborizations from the BraVa database (Wright *et al.*, 2013). NeuroMorpho.Org has, to date, ∼2500 morphological reconstructions of astrocytes including hippocampal and neocortical astroglial cells of various species—exemplar cells and their morphometric analysis are available in Supplementary Figures S1 and S2. Nevertheless, those astrocytes are structurally incomplete; they merely have arborizations and somatic samples with no endfeet data provided. We believe that these reasons are sufficient to rationalize the absence of domain-specific packages dedicated to astroglial morphometric analysis, visualization and, most importantly, meshing.

A large variety of meshing techniques have been developed to reconstruct polygonal mesh models of neurons and vasculature from their morphological representations. These techniques can be classified into three principal categories. The first is concerned with creating mesh models that are convenient for visual analytics. Depending on the objective, these meshes could be highly tessellated if being used for single cell visualization and analysis, or low tessellated when a visualization of full-compartmental simulation of large scale circuit is essential (Eilemann *et al.*, 2012, 2017; Markram *et al.*, 2015). Although they might have various tessellation levels, these meshes are accurate enough to capture all the details of a given morphology, however, they are not required to be watertight (Brito *et al.*, 2013; Garcia-Cantero *et al.*, 2017; Lasserre *et al.*, 2012). The second category creates visually-appealing, or artistic, mesh models that are primarily used for neuroscientific multimedia generation and content creation. These meshes are neither necessarily accurate, even if they are highly tessellated for UV mapping, nor watertight; they just need to be visually realistic with smooth branching to create photorealistic scientific content. These meshes can be created for instance with skin modifiers (Abdellah *et al.*, 2018). The last category is focused on creating high fidelity and optimized watertight mesh models that can be used to establish tetrahedral volumetric meshes for computationally-intensive reaction–diffusion modeling experiments such as Ca^+2^ signaling and cerebral blood flow simulations (Hepburn *et al.*, 2016). The performance of this category is typically non-interactive, where a single mesh could be generated in several seconds, minutes or even a few hours depending on the complexity of the morphology graph (Abdellah *et al.*, 2020; McDougal *et al.*, 2013; Mörschel *et al.*, 2017).

To the best of our knowledge, we found no existing methods or software packages that could be used to build realistic astrocytic mesh models from their morphological descriptions, including endfeet data. We, therefore, build upon the recent work on synthesizing astroglial morphologies (Zisis *et al.*, 2021) and present a robust method capable of reconstructing those missing astrocytic meshes.

### 1.2 Contribution

Our contributions are summarized in the following points:

- A robust and systematic algorithm for reconstructing polygonal mesh models of astroglial cells from their morphological structures, using metaball skinning.
- The algorithm incorporates a hybrid approach to integrate somatic surfaces that are reconstructed on a physically-plausible basis (Abdellah *et al.*, 2017).
- Our implementation is integrated in NeuroMorphoVis, an open source and domain specific package for neuronal analysis, visualization and meshing, to make it accessible to the neuroscientific community.
- We adapted open source mesh optimization (Yu *et al.*, 2008) and mesh repair (Attene, 2010) libraries to be able to reconstruct adaptively tessellated, optimized and watertight mesh models with clean topology.
- An efficient pipeline for creating polygonal astrocytic meshes for visualization and simulation purposes.

## 2 Materials and methods

**Fig. 1.**
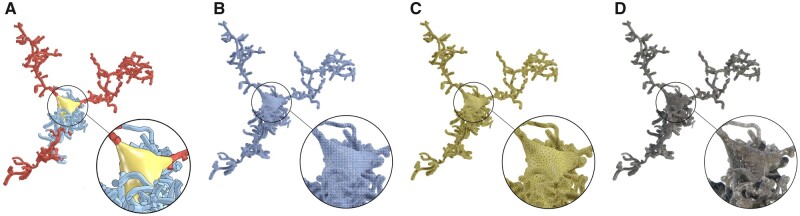
Our pipeline takes synthetic astroglial morphologies (**A**) and create highly tessellated polygonal surface meshes using metaball skinning (**B**), which are adaptively optimized and repaired to produce watertight models (**C**) that can be then used to create volumetric or tetrahedral meshes (**D**) for reaction–diffusion simulations. A detailed structure of the pipeline is illustrated in Supplementary Figure S3

Our core pipeline, illustrated in Figure 1, is composed of three principal stages: (i) *astrocyte morphology skinning*, which produces a surface mesh representing the astrocytic cell membrane, (ii) *astrocyte mesh optimization*, which creates *adaptively* tessellated models for either large scale visual analytics or simulation and (iii) *astrocyte tetrahedralization*, which creates a corresponding volumetric mesh for reaction–diffusion simulations. The astrocytic morphologies are digitally reconstructed with a different pipeline (Zisis *et al.*, 2021) in an earlier stage of our ecosystem.

### 2.1 Astrocyte composition and its morphological structure

**Fig. 2.**
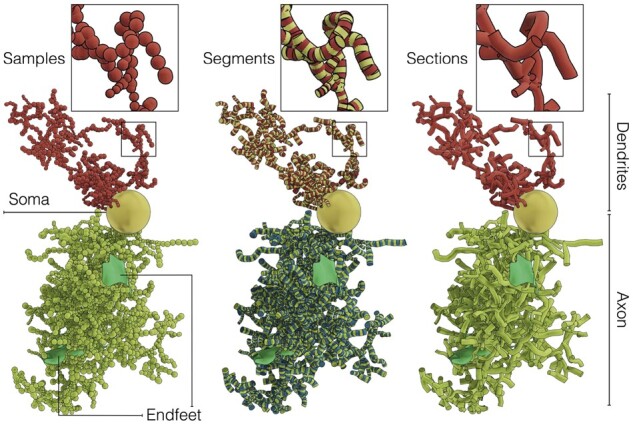
A schematic view of a synthetic cortical astroglial morphology. The skeleton is structured into three principal components: (i) the cell body or soma—in yellow, (ii) the astrocytic neurites and (iii) the endfeet—in green. The neurites—in red—are classified into dendrites (or perivascular processes) and axons (or perisynaptic processes)––in light green. The neurites are composed of morphological samples that are digitally-reconstructed at specific Cartesian locations. Each connected pair of samples defines a segment. A list of connected segments between two branching points defines a section. The sections are arranged in acyclic graphs, where each of them represents an astrocytic neurite. Note that only one dendrite and one axon are shown to avoid clutter, but the full sponge-like structure of the morphology is illustrated in Supplementary Figures S5, S6 and S7

Cortical astroglial cells are similar to neurons; they can be dissected into somata and arborizations with additional endfeet which allow them to interface with the perimeters of cerebrovasculature vessels. Our astrocytic morphologies, refer to (Zisis *et al.*, 2021), are structured into two principal components: (i) a list of connected samples that represent their somata and arborizations and (ii) a list of manifold surfaces that represent the spatial extent of the endfeet. Each sample defines a point in the Cartesian space with a specific diameter and process type. The connectivity information of the sample is obtained with a reference to a parent node and a child one, where a sample with no parent indicates the soma and samples with no children are considered terminals. Excluding the soma sample, each segment in the morphology is composed of two consecutive samples, in which a list of connected segments between two bifurcation points defines a section. An arbor emanating from the soma is represented by a group of sections in an acyclic graph. The endfeet are defined by explicit manifold surfaces whose data are stored as a list of vertices and triangles, where every vertex has a corresponding thickness. The astrocytic morphology structure is illustrated in Figure 2.

### 2.2 Astrocyte skeleton generation

To generate astrocytic morphologies at micrometer resolution, an NGV network model comprised of neurons (Markram *et al.*, 2015; Ramaswamy *et al.*, 2015), astrocytes and vasculature, is digitally reconstructed. Initially, astrocytic somata are placed in the circuit based on experimental cortical density profiles and nearest neighbor distributions. Tiling micro-domains (or bounding polygons) are then generated to determine the accessible region for each astrocyte to grow. From these regions, the synapses and vascular sites are determined. Using neuronal synapses as attraction seeds, astrocytic skeletons are then synthesized based on a hybrid method that combines space colonization and topological branching (Zisis *et al.*, 2021). Constrained by their microdomain region, each astrocyte explores the permissible space to grow toward synaptic points, generating branching patterns according to branching topologies that were extracted from biological experimental reconstructions. The astrocytes project branches to their adjacent vessels to establish the communication pathways between neuronal synapses and the vascular geometry. From the vessel connections, endfeet manifolds are isotropically generated along the vascular surface, simulating the geometry of the perivascular endfeet, which wrap around and tile the vascular walls. Each perivascular branch in the astrocytic skeletons is coupled with an endfoot mesh geometry that apposes the facing vascular surface. A rendering of the vasculature dataset used to synthesize astrocytic skeletons is shown in the Supplementary Figure S4.

### 2.3 Metaball skinning of astrocytic morphologies

Contrary to polygonal meshes represented by vertices and triangular patches, metaballs are implicit surfaces defined procedurally using simple mathematical kernels that can be computed on-the-fly (Karlsson *et al.*, 2019; Oeltze and Preim, 2004; Zoppè *et al.*, 2008). The fundamental advantage of using metaballs in modeling is the ability of multiple complex meta-objects to blend into a single object that is eventually polyganized into a mesh model and cannot be modeled otherwise (Pan *et al.*, 2018). Metaballs are extremely effective, however relatively slow, to reconstruct mesh models of branching structures that are represented either by cyclic graphs, such as vascular networks (Abdellah *et al.*, 2020) or by acyclic graphs, such as neurons (Abdellah *et al.*, 2018). Metaballs are also powerful to handle intricate branching patterns, for example: branches with acute angles, without creating self-intersecting geometries. This problem can be easily noticed for neurons created with Skin modifiers .

We present a robust algorithm for skinning astrocytic morphologies into high fidelity polygonal mesh models based on metaballs in Blender. Our approach includes a hybrid method capable of integrating highly realistic somata profiles that are generated on a physically plausible basis. Each individual component in the astrocyte morphology, including the soma, arbors and endfeet, is skinned into an independent meta-object. These objects are then blended together, neatly, in a single meta-object that is ultimately polyganized into a smooth polygonal mesh that captures all the details of the given astrocytic morphology.

#### 2.3.1 Meta-object initialization

During the initialization stage, an empty meta-object is created. This object is a base, with which all the meta-objects corresponding to each component in the morphology will be blended. The initial polygonization resolution of this meta-object is set to 1.0. Nevertheless, and to capture all the geometric details of the morphology, the resolution must be set to the radius of the smallest sphere along the astrocytic skeleton, which will be known after processing all the components.

#### 2.3.2 Arbors reconstruction

**Fig. 3.**
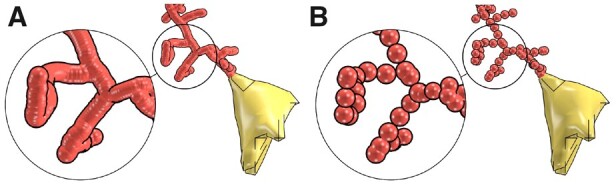
A visual comparison showing a single process of the same astrocytic morphology skeleton before (**A**) and after (**B**) adaptive resampling. Note the amount of unnecessary samples removed. Supplementary Figure S8 provides the same comparison for the entire morphology skeleton

Skinning astrocytic arbors is performed on a per-segment basis; each arbor is skinned into a separate meta-object (or a meta-arbor), each of which is skinned by traversing the hierarchical structure of the arbor—in a depth-first fashion—and operating on each of its segments one-at-a-time. The meta-segments could be, in theory, constructed with the same algorithm for skinning vascular graphs (Abdellah *et al.*, 2020) based on sphere-marching (or metaball marching), where a series of metaballs is generated along the segment axis to interpolate and fill the linear distance between its two samples. However, and due to their synthesis algorithm, astrocytic processes are excessively oversampled. Accessing and processing this large number of samples using the metaball marching kernel is accompanied with a significant performance fall-off. Therefore, it is crucial to adaptively resample each section in the morphology—in a pre-processing step—to preserve its spatial structure while minimizing its number of samples to a convenient extent. Figure 3 highlights the effectiveness of the resampling step on a single astrocytic arbor. Supplementary Figures S9 and S10 show the minimal impact of the resampling process on the structure of the astrocyte morphology using a comparative morphometric analysis before and after resampling.

#### 2.3.3 Somata reconstruction

Typically, and similar to neurons, astrocytic somata are merely described with a single morphological sample that accounts for their origins and mean radii (Brito *et al.*, 2013), see NeuroMorpho.Org. However, superior reconstructions have more samples that project the somatic volume along the optical axis to a two-dimensional profile (Halavi *et al.*, 2008). Likewise, the somata of our synthetic astrocytes are also encoded with the origin-and-radius definition. Accordingly, reconstructing an exact three-dimensional somatic profile is impracticable.

**Fig. 4.**
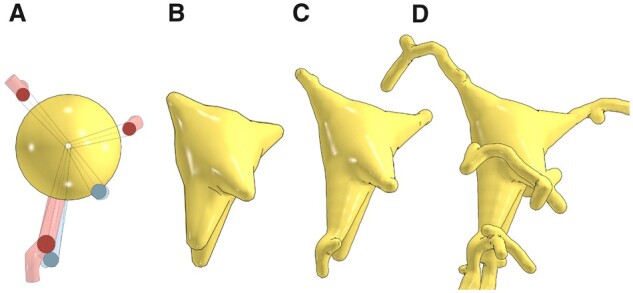
Soma reconstruction with origin-to-arbor metaball marching. The meta-soma is created initially (**A**) and extended toward the different arbors in the morphology (in transparent red and blue) till the somatic profile is completely reconstructed in (**B**). This meta-object is then blended with the counterparts of the arbors (**C**) and (**D**). The reconstructed somatic surface can be slightly altered with different polygonization resolution, see Supplementary Figure S11

Our implementation provides two different approaches to reconstruct approximate somata profiles. We refer to the first approach as the origin-to-arbor metaball marching, which is logically similar to skinning segments. This relatively fast approach is intended to create simplified somatic profiles. Initially, a single metaball (meta-soma), whose center and radius match the somatic sample, is created. The connectivity between the soma and the arbor is considered as a segment (somatic segment), whose terminal samples are the origin of the soma and the initial sample of the corresponding arbor. For each arbor, the respective somatic segment is interpolated and filled with a series of metaballs. The meta-soma and meta-segments are then all blended to form the final soma profile as illustrated in Figure 4.

**Fig. 5.**
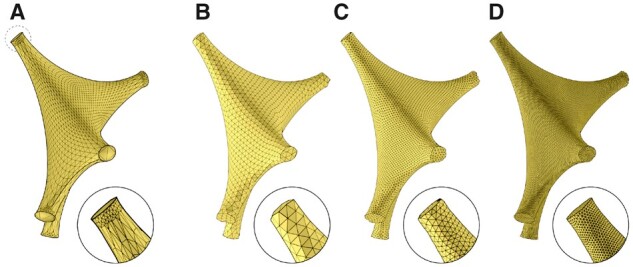
The point cloud (or vertices) of the initial surface mesh obtained from ordinary soft body simulation in NeuroMorphoVis is not uniformly distributed (**A**). We, therefore, use a particle system remesher to create uniformly tessellated surface mesh to be used for skinning the somatic surface with metaballs. The resulting mesh is subdivided with one (**B**), two (**C**) and three (**D**) subdivision levels

The second approach hybridizes our metaball skinning technique with previous methods for building physically plausible profiles (Abdellah *et al.*, 2017; Brito *et al.*, 2013) with Hooke’s law and mass-spring models. This hybridization assumes that a uniformly sampled point cloud of the somatic surface with sufficient sampling exists, with which we can create meta-patches covering the entire surface. Those patches can be generated by creating a metaball for each individual point across the surface. If the maximum distance between one point and the surrounding points in this cloud is smaller than the polygonization resolution of the base meta-object, those patches are then guaranteed to blend smoothly with the meta-arbors in order to yield continuous and flat connections between the soma and the respective arbors. Since we implement our algorithm in NeuroMorphoVis (Abdellah *et al.*, 2018), we create an initial surface manifold that represents a realistic approximation of the somatic profile using the soma reconstruction toolbox. Unfortunately, due to the pulling forces that are applied on the soft body model to create this surface, its vertices are not uniformly distributed across its surface, see Supplementary Figure S12. To resolve this constraint, we remesh this surface with a particle-based meshing algorithm that guarantees the creation of polygonal meshes with uniformly distributed vertices as shown in Figure 5 and Supplementary Figure S13. The vertices of this remeshed surface are then used to create the meta-patches necessary to define the final shape of the meta-soma, which is ultimately used to blend with the rest of the meta-components. For each vertex in this surface, a metaball will be created until the entire mesh is covered, see Supplementary Figure S14.

**Fig. 6.**
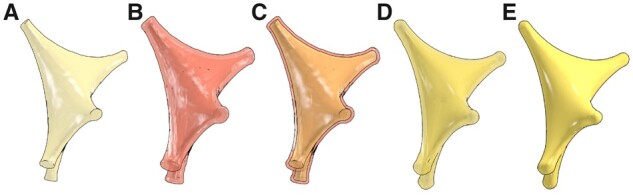
Due to the nature of the metaball algorithm, the resulting somatic surface is composed of two mesh partitions having different volumes and surface areas. If we build a somatic surface using the input mesh shown in (**A**), we will get a resulting mesh with two partitions (**B**), that cover the input mesh as shown in (**C**). The displacement step is used to ensure that the exterior partition has an approximate volume to that of the input mesh (**D**), where we can eliminate the interior partition to end up with a somatic surface with a single partition matching the input one (**E**)

**Fig. 7.**
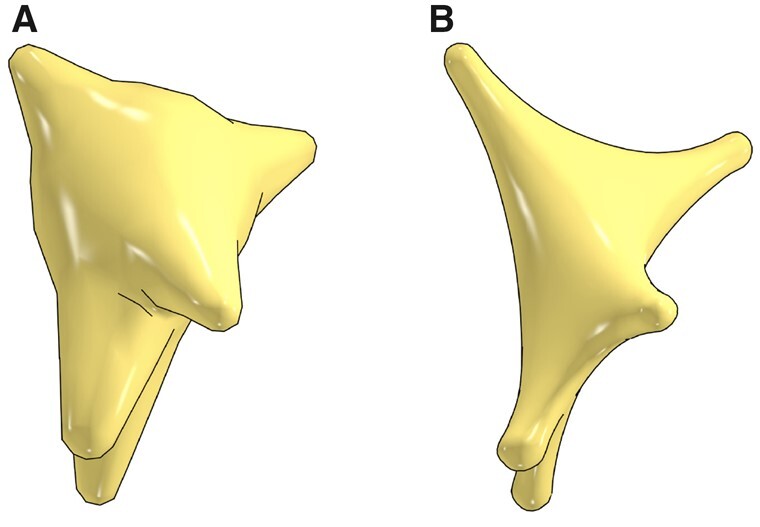
Three-dimensional somatic profiles for the same astrocyte reconstructed with the origin-to-arbor metaball marching method (**A**) and the hybrid method (**B**)

If the meta-patches are blended and polygonized, two mesh surfaces (or partitions) will be created. These partitions are surrounding the input manifold surface that is used to create the meta-patch as shown in Figure 6. Obviously, these partitions have different volumes and surface areas in comparison with the original mesh that was generated in Figure 5A. Therefore, we tweak our algorithm to displace the vertices of the original mesh with a step that is computed based on the radius used to build the metaball and the normal of the vertex. Doing so, we can use only the external partition and remove the other one from the mesh. This minor adjustment was sufficient to create somatic profiles with approximate volume and surface areas as shown in Figure 6E. The somatic surfaces generated with the two approaches are comparatively shown in Figure 7.

#### 2.3.4 Endfeet reconstruction

Endfeet data are not given as part of the morphology hierarchy per se, but rather it is retrieved indirectly from the NGV circuit that defines the connectivity between astroglia, neurons and vasculature based on their astrocyte identifier in the circuit. Each astrocyte has one endfoot or more, where each is represented by an open surface that is a two-dimensional manifold. This surface is defined by a connected patch of triangles and vertices, where each vertex defines a three-dimensional Cartesian coordinate and a diameter that reflects the thickness of the endfoot at this point in space. Although surface normals are not given across the patch, they are not needed to skin the endfeet as opposed to somata.

**Fig. 8.**
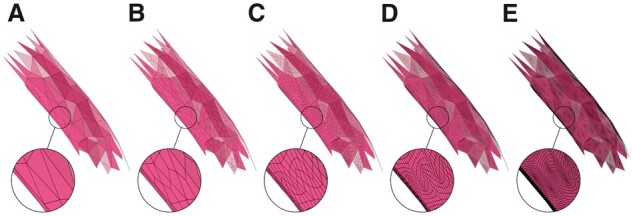
The endfeet are subdivided to avoid creating fragmented partitions around their extent. The input endfoot patch in (**A**) is subdivided with one (**B**), two (**C**), three (**D**) and four subdivision levels. By trial-and-error, subdivision level of three was found to be reasonable

Starting from those triangular patches, we present a simple, yet efficient, approach to skin endfeet manifolds into multiple meta-objects. A given endfoot patch cannot be processed directly to build a corresponding meta-object due to the limited tessellation of the patch with respect to the endfoot thickness, which means that the average distance between the vertices of each triangle across the endfoot surface is greater than the thickness per vertex. Therefore, if those vertices are used to reconstruct the meta-object, it will merely create a group of fragmented mesh partitions, as shown in Supplementary Figure S15. This issue is resolved by increasing the tessellation of the patch using the surface subdivision operator, as shown in Figure 8. The tessellation factor is set based on the smallest thickness across the endfoot surface. We added the capability to select between two subdivision types: (i) simple subdivision, which increases the patch density without any surface smoothing, or (ii) Catmull–Clark subdivision, which subdivides and smooths the surface to focus on the esthetic appearance of the endfoot as indicated in Supplementary Figure S16. After applying the subdivision operator, the resulting patch is conveniently processed and converted into a meta-object that when polygonized, will create a smooth surface with no holes or open patches.

#### 2.3.5 Meta-objects polygonization, or meshing

After the skinning of each component in the astrocytic morphology into a separate meta-object, all the meta-objects are seamlessly blended together into a single meta-object for polygonization or meshing. This process is implemented under the hood in Blender based on one of the variants of the marching cubes algorithm. The final resolution of the meta-object is therefore set to the radius of the minutest sample in the entire morphology prior to polygonization to avoid creating a fragmented mesh.

As discussed earlier, and depending on the approach used to create the somatic meta-object, the resulting mesh will have at least one or at most two partitions. If the origin-to-arbor metaball marching method is used, it is guaranteed that the mesh will only have a single partition. If the hybrid approach is used, the astrocytic mesh will have two partitions. In the latter case, the mesh is further post-processed to eliminate the unwanted partition, which has less polygons and or vertices. The resulting mesh might be highly tessellated, depending on the minimum radius in the morphology skeleton, but it is guaranteed to have zero non-manifold edges and vertices.

### 2.4 Astrocytic mesh tessellation and optimization

The astrocytic meshes created with our metaball skinning algorithm are ordinarily highly tessellated, which could potentially limit their usability—for instance, to load thousands of them at once to visualize the connectivity of a multi-populated NGV circuit for verification. In case of being used for reaction–diffusion simulation, these meshes might not be convenient for tetrahedralization due to their immoderate size. Consequently, it is necessary to optimize and re-tessellate these meshes in an adaptive fashion.

The meshes can be selectively decimated using the default subdivision library that is integrated in Blender into lightweight meshes that are almost a tenth of the size or even less—with no significant loss in their spatial extents. This subdivision approach is convenient for obtaining meshes that can be used for large scale rendering, where we need to visualize a large number of astrocytes all at once to verify their locations with respect to the vascular network. Nevertheless, these decimated meshes do not have clean topologies and might have self-intersections, and therefore, they cannot be used for performing reaction–diffusion simulations. For this purpose, we have adapted a couple of open source frameworks for mesh optimization (Yu *et al.*, 2008) and repair (Attene, 2010) to ensure that our pipeline is capable of creating optimized watertight mesh models that can be tetrahedralized with existing tetrahedralization solutions.

### 2.5 Tetrahedralization

Tetrahedralization converts a two-dimensional closed manifold represented by a polygonal surface mesh into a three-dimensional volumetric mesh containing multiple tetrahedra. This process is essential to build a geometric astrocytic model that can be used to perform reaction–diffusion simulations (Hepburn *et al.*, 2016). Nevertheless, tetrahedralization cannot be accomplished in a reasonable time without the existence of a watertight mesh that is by definition two-manifold and has no self-intersections. Implementing a tetrahedral mesh generator is beyond the scope of this work, and therefore we have relied on a convenient solution to synthesize tetrahedral astrocytic models to complement our pipeline. Various open source implementations and Application Programming Interfaces (APIs) are already available, for example TetGen (Si and TetGen, 2006), CGAL (Boissonnat *et al.*, 2000) and Quartet (Labelle and Shewchuk, 2007). For convenience, we have extended the Quartet implementation and integrated it into our pipeline to create tetrahedral meshes that are stored in the GMesh file format (Geuzaine and Remacle, 2009). It has to be noted that a recent tetrahedral meshing technique called TetWild is capable of creating tetrahedral models from non-watertight meshes that might have self-intersections and a few small holes (Hu *et al.*, 2018). Nevertheless, the implementation of the technique is relatively slow and has high memory footprint; it cannot be used to tetrahedralize an astroglial cell with complex arborizations at convenient spatial resolution sufficient to perform reaction–diffusion simulations.

## 3 Results and discussion

### 3.1 Implementation

Our skinning algorithm is implemented as an add-on based on NeuroMorphoVis and the Python API of Blender. The mesh optimization and tetrahedralization parts are implemented in C++, but their executables are invoked within the Python code. The add-on can be executed either from a CLI or using a configurable shell script, refer to Section II in the Supplementary Material. To increase its throughput, the pipeline is parallelized relying on the JobLib package to allow running multiple instances on a single compute node. However, and for convenience, users can manually set a specific number of cores for batch processing in case of memory limitations.

### 3.2 Running the pipeline

**Fig. 9.**
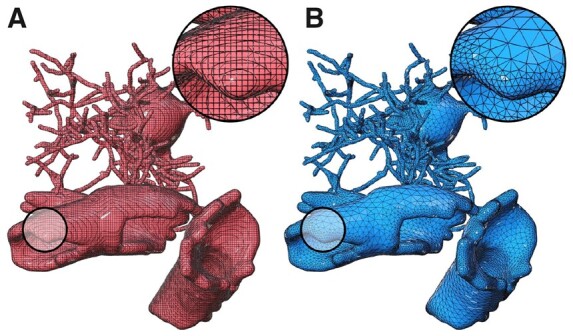
Wireframe models of the resulting meshes from our pipeline. (**A**) The mesh is generated with the skinning implementation without any decimation. (**B**) The mesh in (A) is optimized and adaptively tessellated to have clean topology

The pipeline was tested with 5000 astrocytic morphologies that were synthesized with a recent NGV multi-populated circuit. It was configured to generate two sets of polygonal surface meshes: (i) those produced directly from the skinning implementation at full tessellation and (ii) a set of optimized and adaptively tessellated ones. The qualitative geometric metrics of both meshes are evaluated to validate their accuracy (Knupp *et al.*, 2006). Figure 9 shows a side-by-side comparison between the non-decimated mesh and its optimized counterpart for an exemplar astrocytic morphology. This comparative analysis is reported for nine more pairs in Supplementary Figures S17–S25 in addition to their corresponding qualitative metrics in Supplementary Figures S26–S34. The pipeline was executed in parallel on a compute node having 72 cores, but only 15 of them are used. The 5000 meshes were created in nearly 4 h and stored in. OBJ file format.

### 3.3 Meshes for visual analytics

**Fig. 10.**
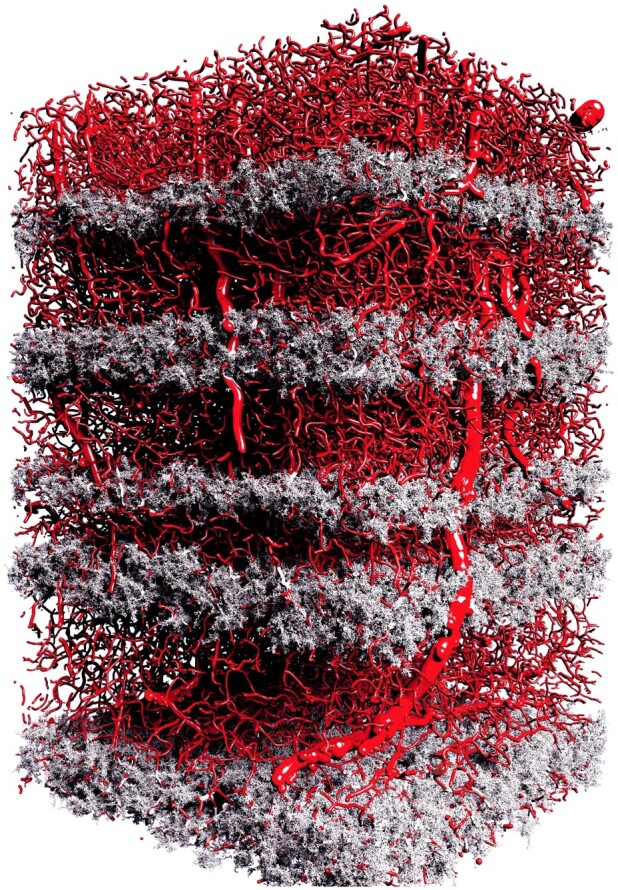
A high-quality rendering of the vasculature mesh shown in Supplementary Figure S4 combined with the 5000 decimated astrocytic meshes generated with our pipeline. The total size of astrocytic meshes is ∼20 GB. The image is rendered with the OSPRay rendering engine that is integrated in Brayns. This rendering is used to verify the correct placement of the astrocytes in the NGV circuit. The rendering is available in higher resolution in Supplementary Figure S35

**Fig. 11.**
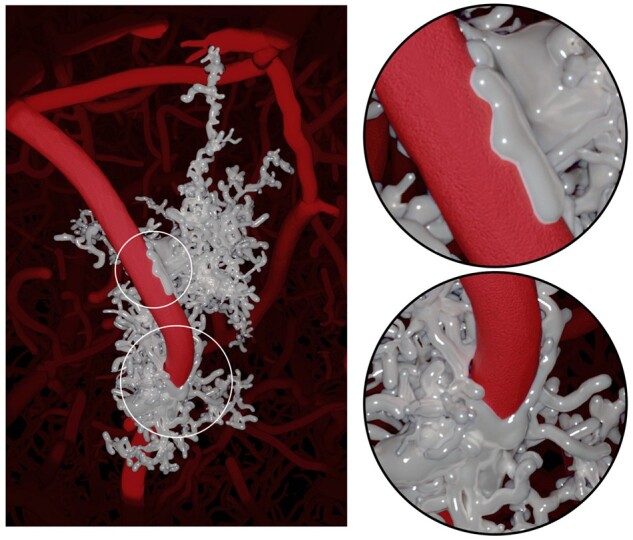
The non-decimated meshes are used to verify astrocytes placement and their connectivity to vasculature, where endfeet wrap around blood vessels. The image is rendered in Blender using Cycles, and is available in higher resolution in Supplementary Figure S34

We further created from the first set of meshes a group of low-tessellated ones decimated at 10%. We then used Brayns, a neuroscience specific large scale and interactive visualization system, to visualize the structural composition of the NGV circuit and the spatial relationships between the astrocytes, in addition to their connectivity to the vascular graph as shown in Figure 10. We also used the non-decimated meshes to visualize how the perivascular endfeet are connected and wrapped around the blood vessels to verify if they intersect with the vascular wiring or not, as illustrated in Figure 11.

### 3.4 Visualizing reaction–diffusion simulations

**Fig. 12.**
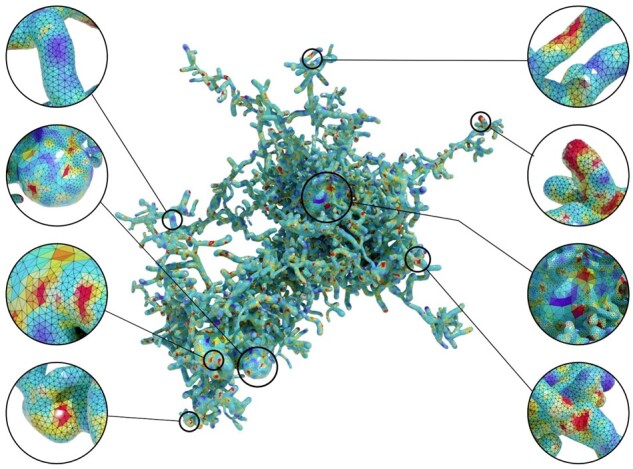
A tetrahedral mesh of an exemplar astrocyte showing a randomly generated simulation report to mimic the variations of Ca^+2^ concentrations across the astrocytic surface. This tetrahedral mesh is created with QuarTet using an input surface mesh reconstructed with our metaball skinning add-on. A higher resolution of this image is available in Supplementary Figure S37

In fact, performing stochastic reaction–diffusion simulations are beyond the scope of this work. However, it was important to verify the usability of the optimized meshes created with our pipeline. We, therefore, developed a visualization prototype capable of reading simulation reports, such as Ca^+2^ concentrations, generated with STEPs—a scientific package for simulating reaction–diffusion systems in realistic tetrahedral mesh models of biological structures (Hepburn *et al.*, 2012). In our context, such simulations permit investigations of the time course and temporal mixing of signals at multiple sites within the astrocyte geometry, where Ca^+2^ ions are released. Accurate realizations, however, require incorporating detailed models of other intracellular organelles such as the endoplasmic reticulum (ER), which has yet to be modeled in our NGV circuit. Consequently, we had to generate a time series of random concentration data to visualize their variations within the entire cell extent over time. Figure 12 shows a tetrahedral mesh model of an exemplar astrocyte synthesized from an optimized astrocytic polygonal surface mesh at a single time step. Supplementary Figure S38 shows multiple snapshots of the simulation at different time steps.

### 3.5 General remarks

Users can selectively build somatic surfaces with either of the approaches illustrated in Figure 7. Obviously, the hybrid approach creates highly realistic somata compared to the naïve one. Moreover, the soft body parameters, including stiffness and simulation steps, can be tuned to improve the realism of the reconstructed profiles, see Supplementary Figure S39. Nevertheless, this approach is relatively slow; for example, it takes with this approach ∼28 s to reconstruct a full astrocyte mesh, compared to 1.8 s for the naïve method. This concern is partially resolved by parallelizing the pipeline. Users can also select whether to produce meshes for visualization, simulation or for both aspects. The decimation does not impact the simulation mesh; it is merely used to create low-tessellated visualization-specific meshes where we can load thousands of them in a single scene to visualize a full circuit. This does not mean that those meshes are inaccurate; even with a decimation factor of 0.1, the RMS value of the Hausdorff distance (Ghaffari *et al.*, 2018) is ∼0.03, which is within acceptable range as shown in Supplementary Figure S40.

### 3.6 Validation and feedback

The output meshes were validated by biologists to ensure that no artifacts were introduced during the reconstruction process. The two parameters most key to biologists, surface area and volume, were assessed in order to understand the tradeoff with resolution. Furthermore, the final meshes were simulated in a reaction–diffusion simulator, thereby validating the robustness of the reconstruction process. Full simulation of the entire system from a biological perspective will be the addressed in our future work. Already biologists in the broader simulation community have shown keen interest in using the astrocyte meshes for their simulations, as no other comparable meshes yet exist.

## 4 Conclusion

Astrocytes play an essential role in forming the physical structure of the brain. They contribute to almost every conceivable brain function and disorder. Unraveling their underlying functional aspects entails the existence of ultrastructurally accurate models that can be used to perform chemical simulations at microscopic resolutions. In contrast to neurons, the literature is lacking detailed biological reconstructions of astrocytic cells. Recent efforts have started to resolve this issue and developed a data-driven approach to algorithmically generate digital astrocytic morphologies that are statistically indistinguishable from biological ones. We developed an unconditionally robust method capable of skinning those astrocytic morphologies to accurate and optimized astrocytic mesh models with realistic geometries. This method is based on implicit surfaces, which makes it possible to skin complicated structures that are impossible to model accurately otherwise. We also presented a hybrid approach that incorporates physically plausible somatic surfaces into our astrocytic models to improve the realism of the reconstructed meshes. On top of that, we designed an end-to-end pipeline, which optimizes these two-dimensional meshes and generates three-dimensional tetrahedral meshes that can be supplied to reaction–diffusion simulators for modeling astrocytic functions.

We used our pipeline to generate 5000 astrocytic meshes to visually analyse their placement and connectivity in the NGV circuit. The pipeline is made available to the neuroscientific community by integrating the implementation into an open source package dedicated to neuronal visualization. Finally, our skinning algorithm is generic and applicable to reconstruct mesh models of biological astrocytic morphologies, not necessarily the synthetic ones, as long as endfeet data are incorporated within the morphology files.

## Software availability

The metaball skinning algorithm is implemented based on the Python API of NeuroMorphoVis (Abdellah *et al.*, 2018), which is freely available under the GNU public license on Github. The implementation was tested with Blender 2.80 and 2.90. The pipeline is executable via CLI and configuration files. The code is open sourced under the regulations of the Blue Brain Project, École Polytechnique Fédérale de Lausanne (EPFL).

## Data sources

The original vasculature skeleton (in.VTK format) that is used to synthesize the astrocytic morphologies is provided by Bruno Weber, University of Zürich (UZH), Switzerland. The astrocytic morphologies are provided by Eleftherios Zisis, Blue Brain Project, École Polytechnique Fédéral de Lausanne (EPFL), Switzerland. The reconstructed astrocytic meshes can be made available from the corresponding authors on a reasonable request.

## Supplementary Material

btab280_Supplementary_Data
