# Supplementary material for: Metaball skinning of synthetic astroglial morphologies into realistic mesh models for in silico simulations and visual analytics
Source: Bioinformatics. 2021 Jul 12;37(Suppl 1):i426–33. doi: 10.1093/bioinformatics/btab280 (PMC8275327; doi:10.1093/bioinformatics/btab280)
Supplement: btab280_Supplementary_Data [file btab280_supplementary_data.pdf]

Supplementary Materials

Metaball skinning of synthetic astroglial morphologies into realistic mesh models for in silico simulations and visual analytics

Marwan Abdellah*, Alessandro Foni, Eleftherios Zisis, Nadir Román Guerrero, Samuel Lapere, Jay S. Coggan, Daniel Keller, Henry Markram and Felix Schürmann*

Blue Brain Project (BBP), École polytechnique fédérale de Lausanne (EPFL), Campus Biotech, Geneva 1202, Switzerland

*To whom correspondence should be addressed.

*marwan.abdellah@epfl.ch and felix.schuermann@epfl.ch

© The Author 2021.

1 Supplementary figures

1.1 Astrocyte morphologies from NeuroMorpho.Org (?)

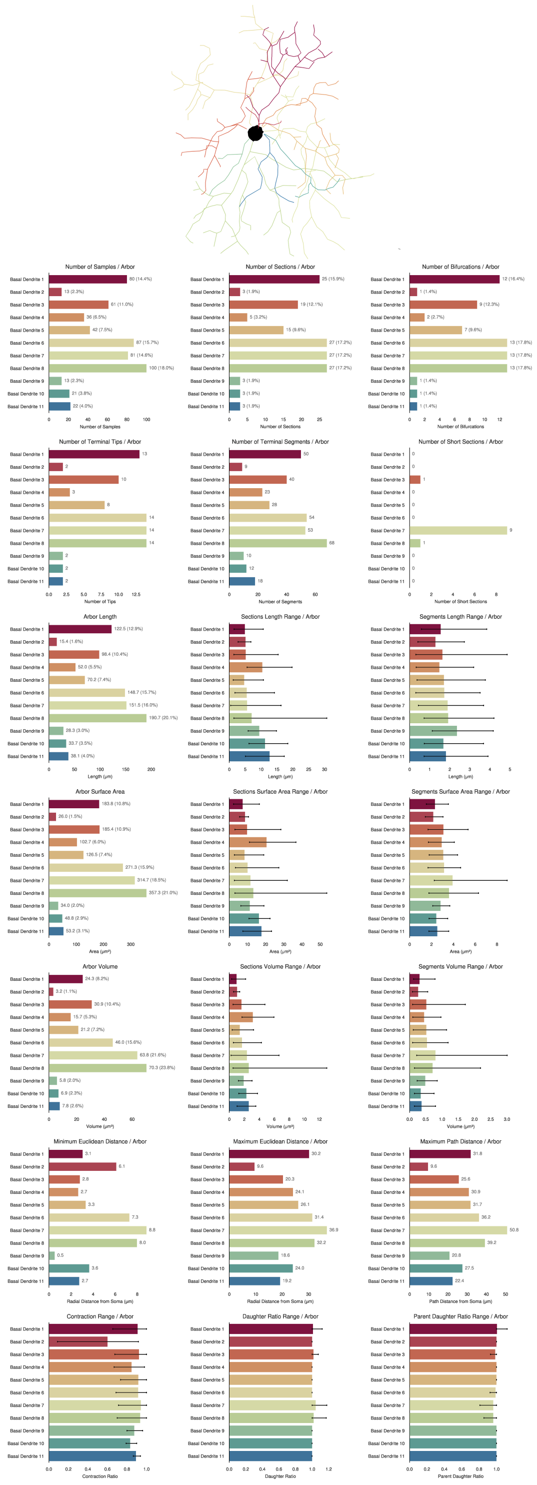

Figure S1. Morphometric analysis of the astrocytic morphology **Ctx-SmoCKO-GFAP-astro1.CNG**. The morphology is available as an .SWC file from NeuroMorpho.Org.

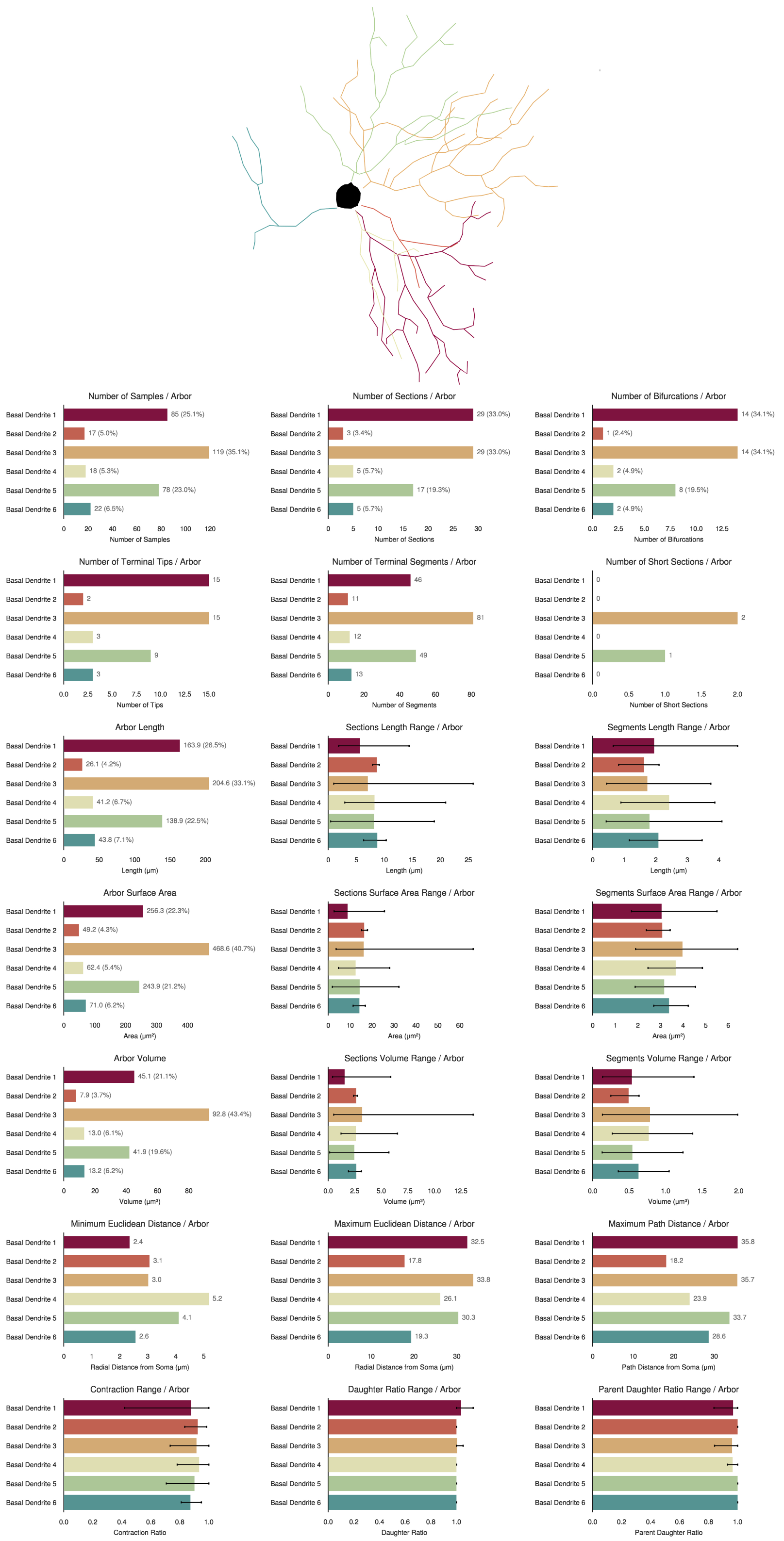

Figure S2. Morphometric analysis of the astrocytic morphology **Ctx-SmoCKO-GFAP-astro8.CNG**. The morphology is available as an .SWC file from NeuroMorpho.Org.

1.2 Mesh generation pipeline

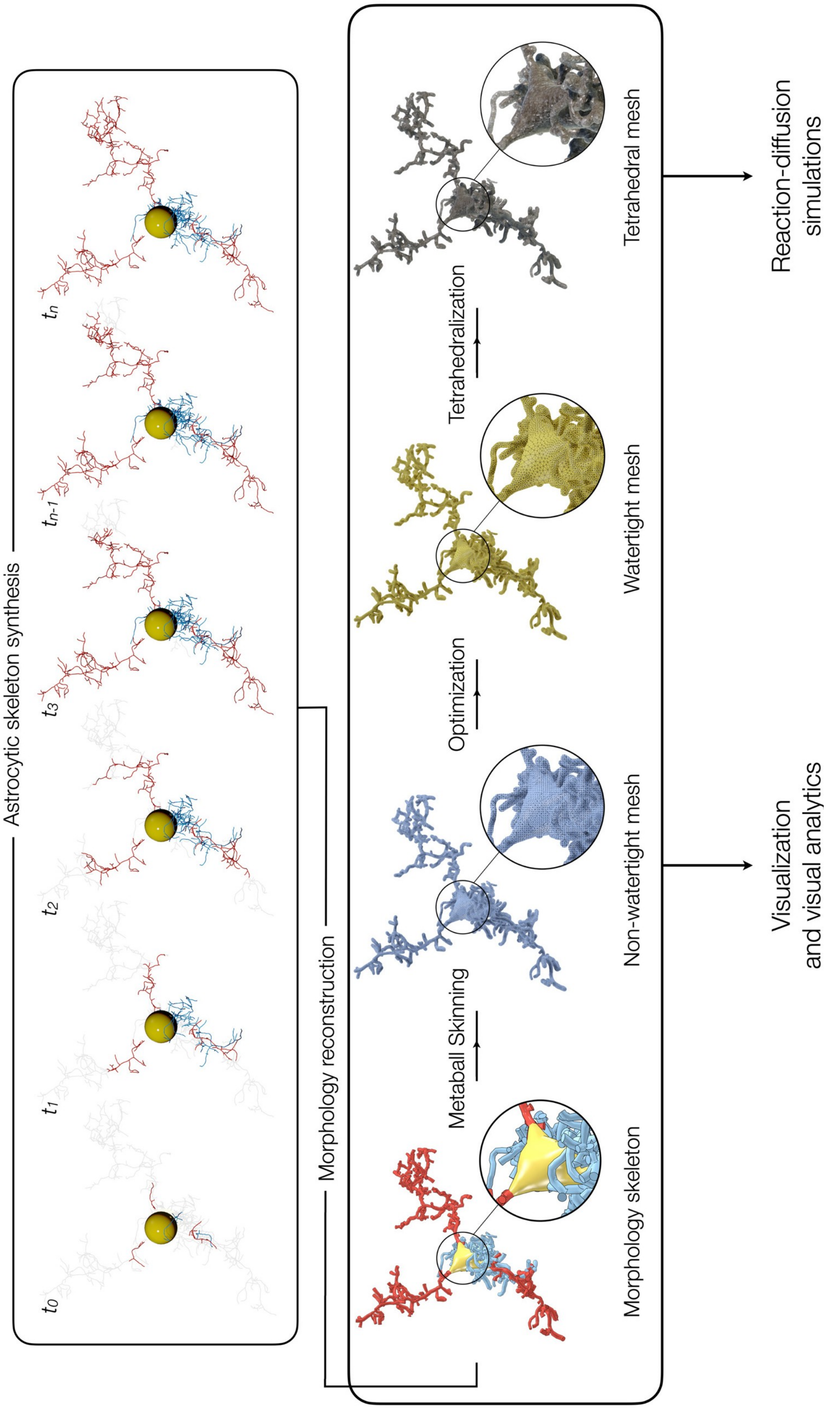

Figure S3. Our pipeline takes synthetic astroglial morphologies (A) and create highly tessellated polygonal surface meshes using metaball skinning (B), which are adaptively optimized and repaired to produce watertight models (C) that can be then used to create volumetric or tetrahedral meshes (D) for reaction-diffusion simulations. Note that the astrocytic morphologies are digitally reconstructed with a different pipeline (?) in a previous stage of our ecosystem.

1.3 Vasculature dataset

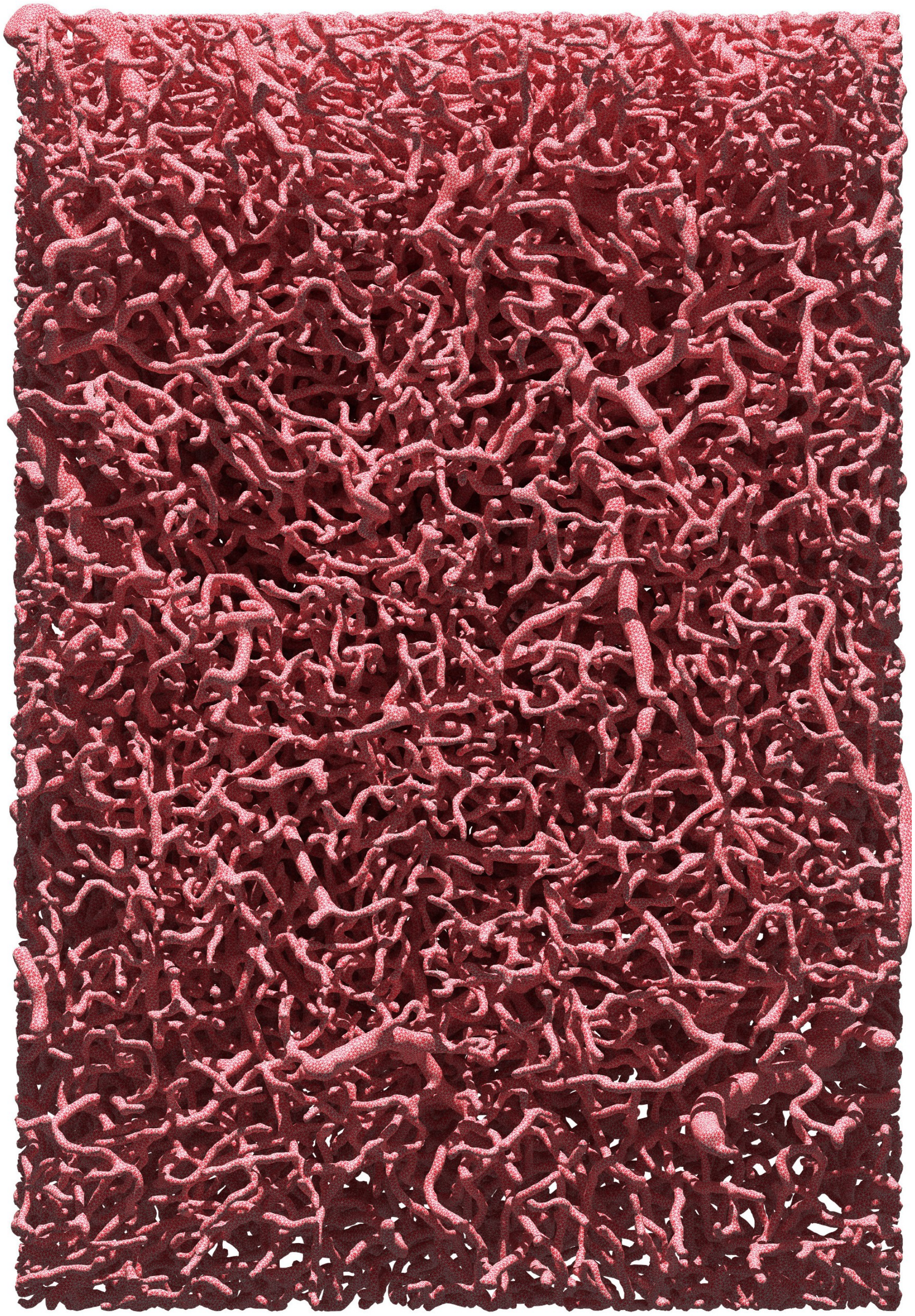

Figure S4. A photo-realistic rendering of the vasculature dataset mesh that is used to synthesize the astrocyte morphologies. This vascular network has the following dimensions: $955 \times 1452 \times 853\ \mu\text{m}^3$ corresponding to a volume of $1.18\ \text{mm}^3$. The original reconstruction (in .VTK format) is provided by Bruno Weber (University of Zurich, Switzerland).

1.4 Astrocytic morphology structure

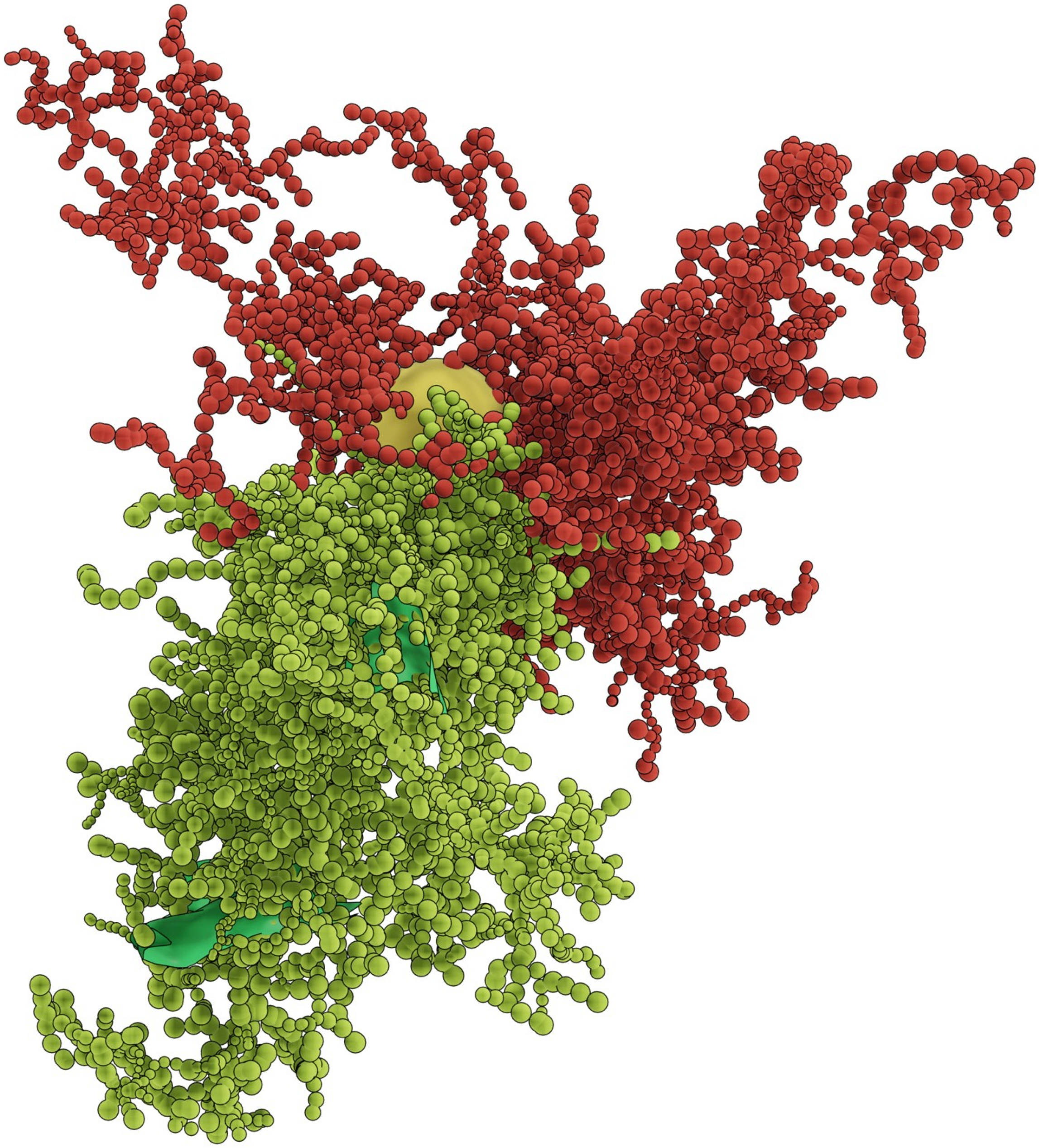

Figure S5. Astrocytic morphology skeleton rendered as list of morphological samples that represent the processes and a list of surface patches that represent the endfeet. The soma and endfeet are shown in yellow and green respectively.

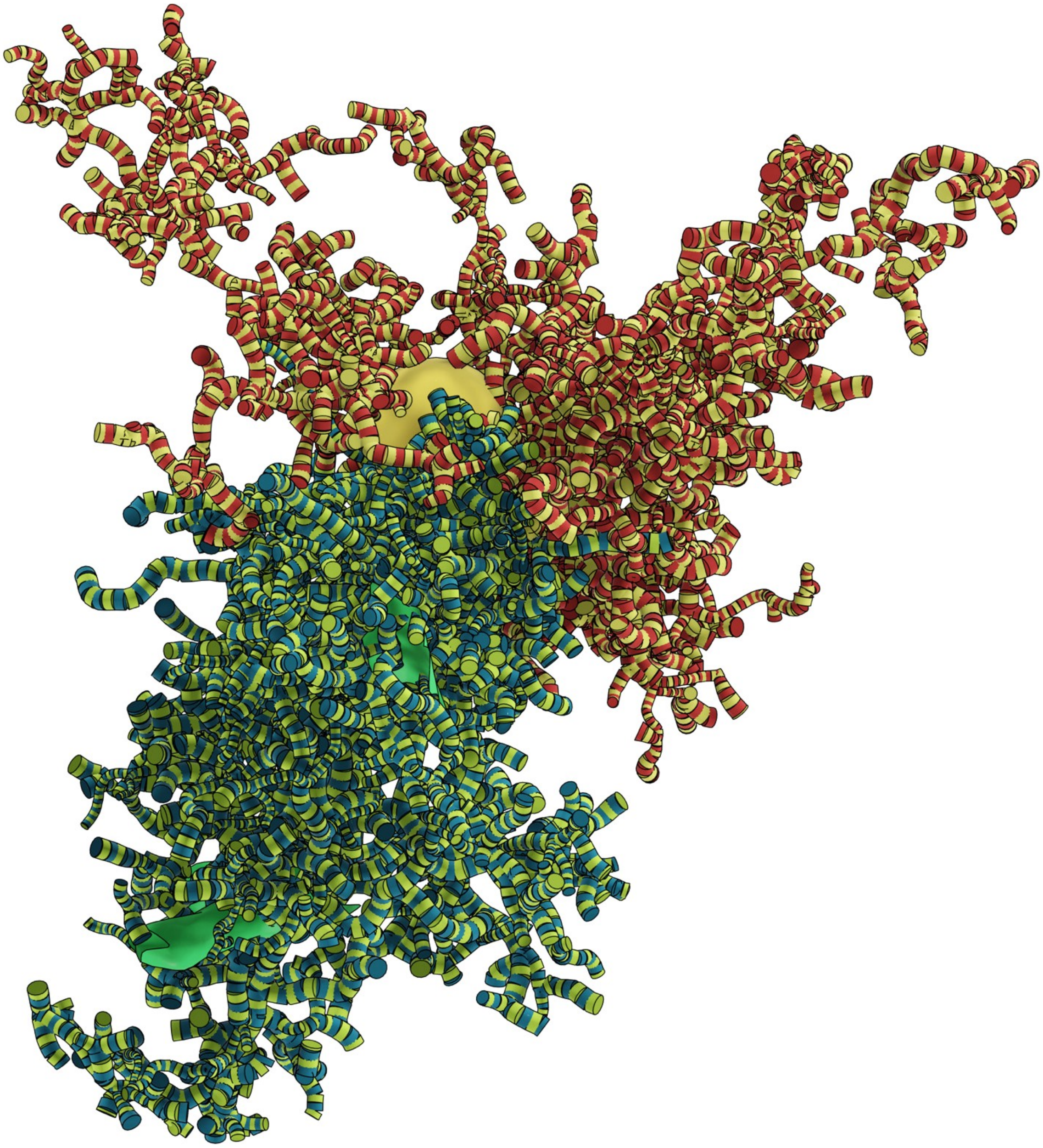

Figure S6. Astrocytic morphology skeleton rendered as list of alternating-color segments that represent the processes and a list of surface patches that represent the endfeet. The soma and endfeet are shown in yellow and green respectively.

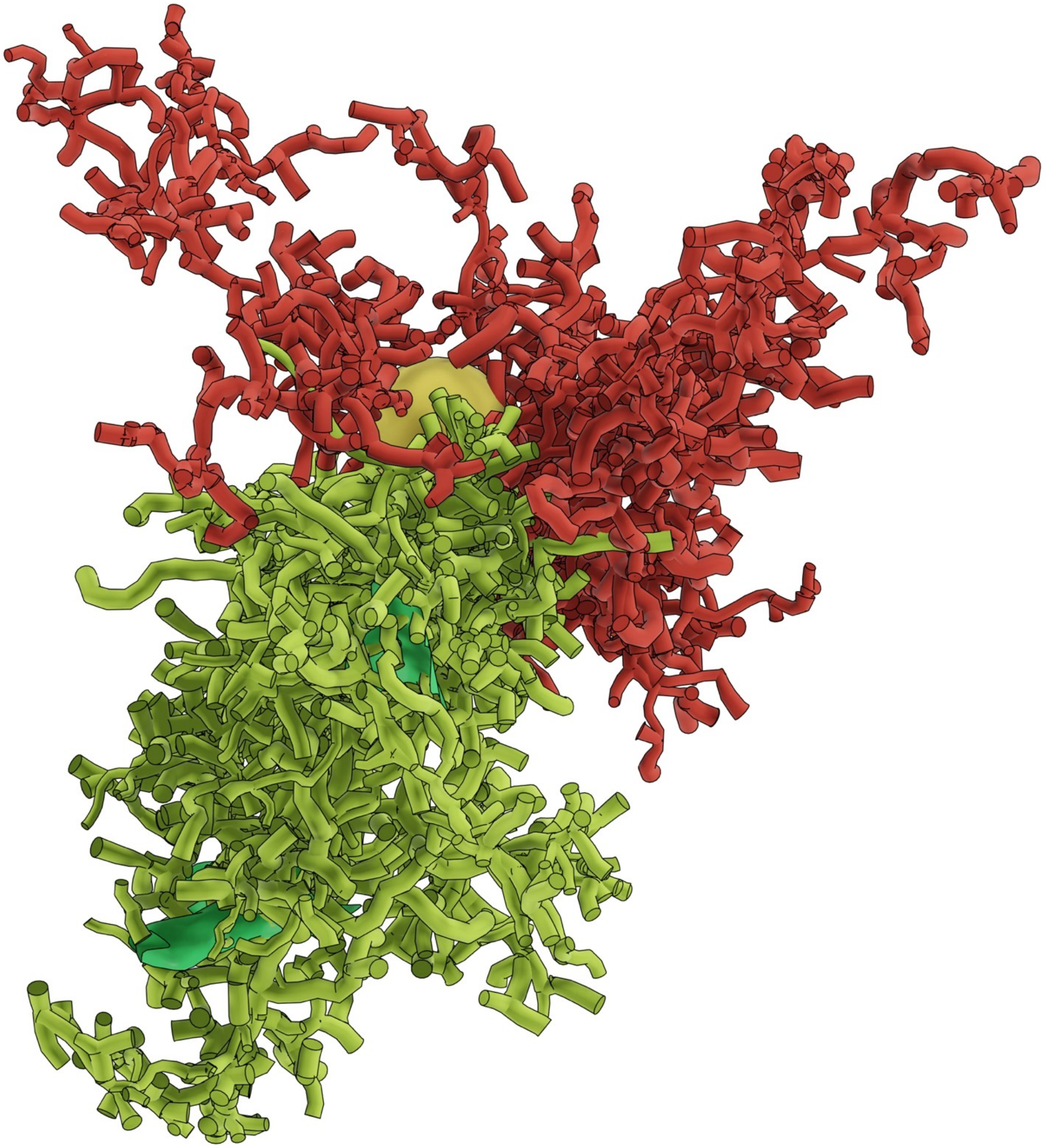

Figure S7. Astrocytic morphology skeleton rendered as list of connected sections that represent the processes and a list of surface patches that represent the endfeet. The soma and endfeet are shown in yellow and green respectively.

1.5 Morphology resampling

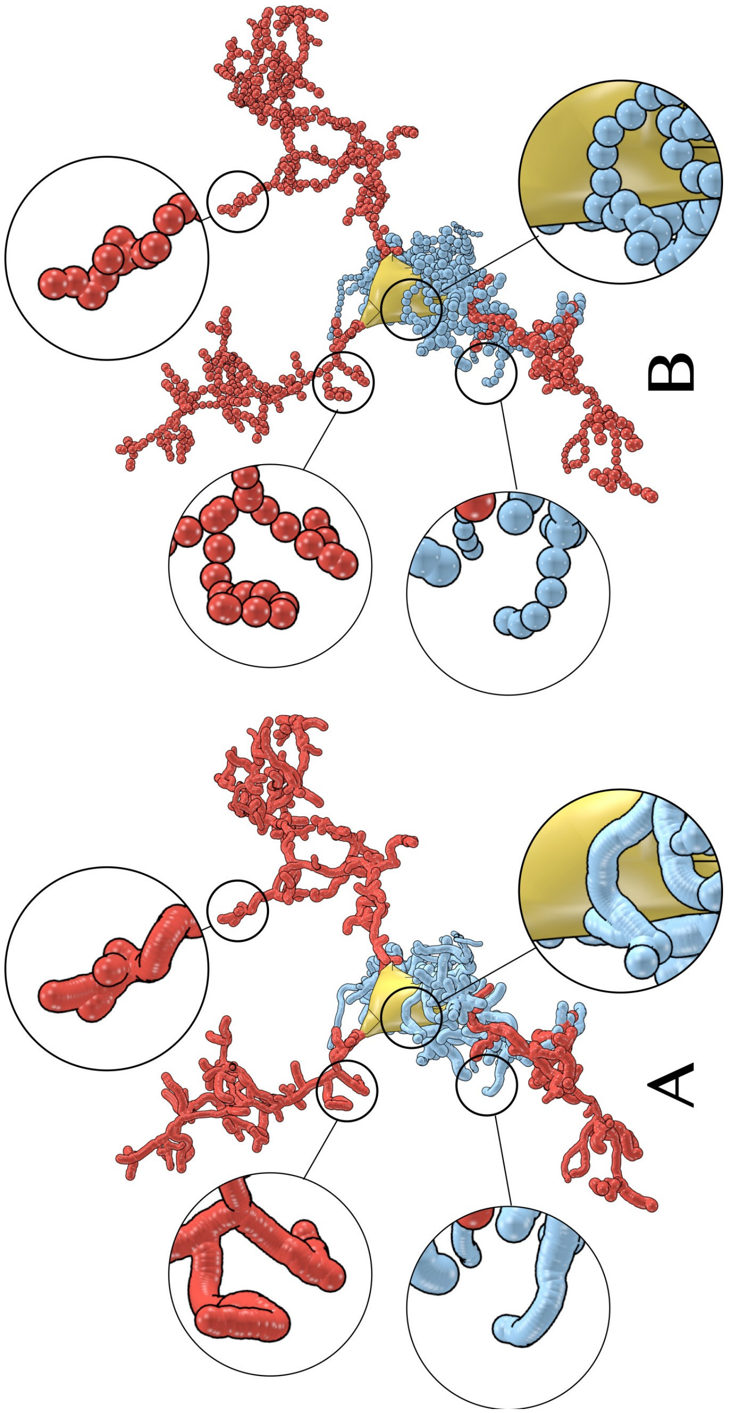

Figure S8. A visual comparison showing the same astrocytic morphology skeleton before (left) and after (right) adaptive resampling

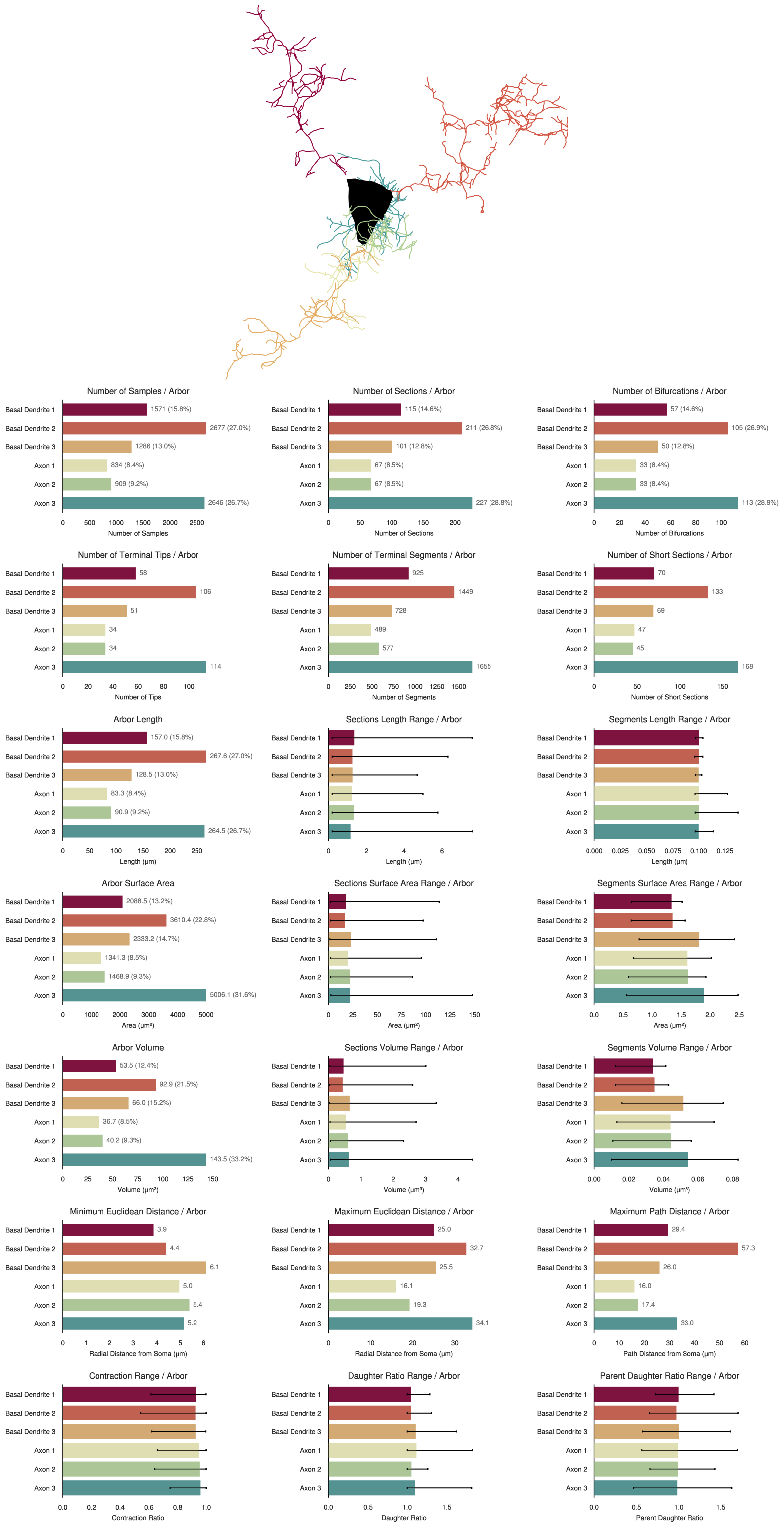

Figure S9. Morphometric analysis of the astrocytic morphology skeleton **before** applying the adaptive resampling kernel.

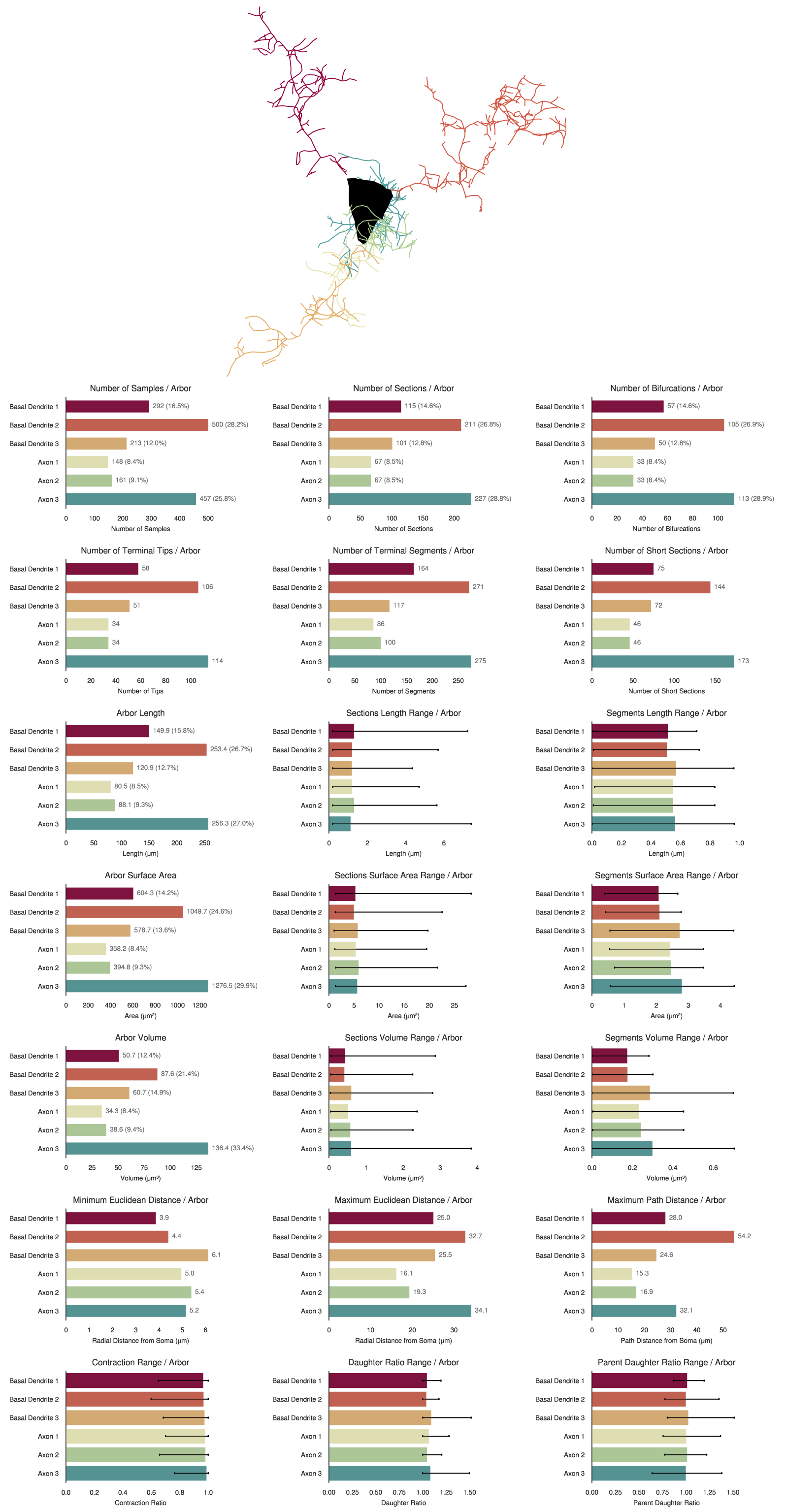

Figure S10. Morphometric analysis of the astrocytic morphology skeleton **after** applying the adaptive resampling kernel. Note the amount of samples compared to Figure S9.

1.6 Soma reconstruction

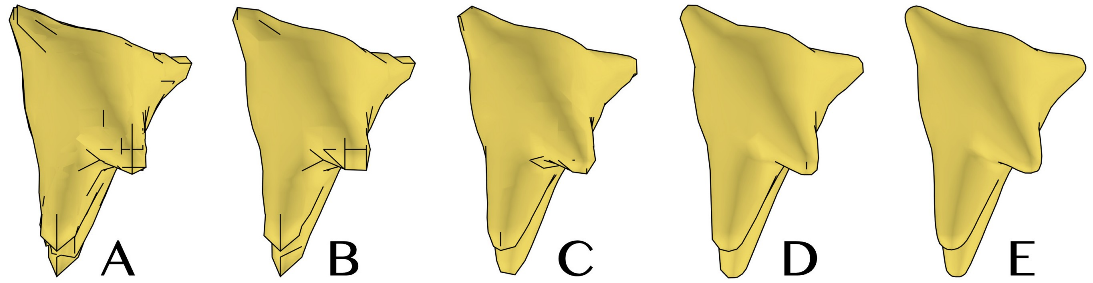

Figure S11. Soma reconstruction with origin-to-arbor metaball marching with different polygonizations resolutions: (A) 0.9, (B) 0.5, (C) 0.25, D (0.1) and E (0.05). Note that the somatic profile does not change significantly with varying the resolution.

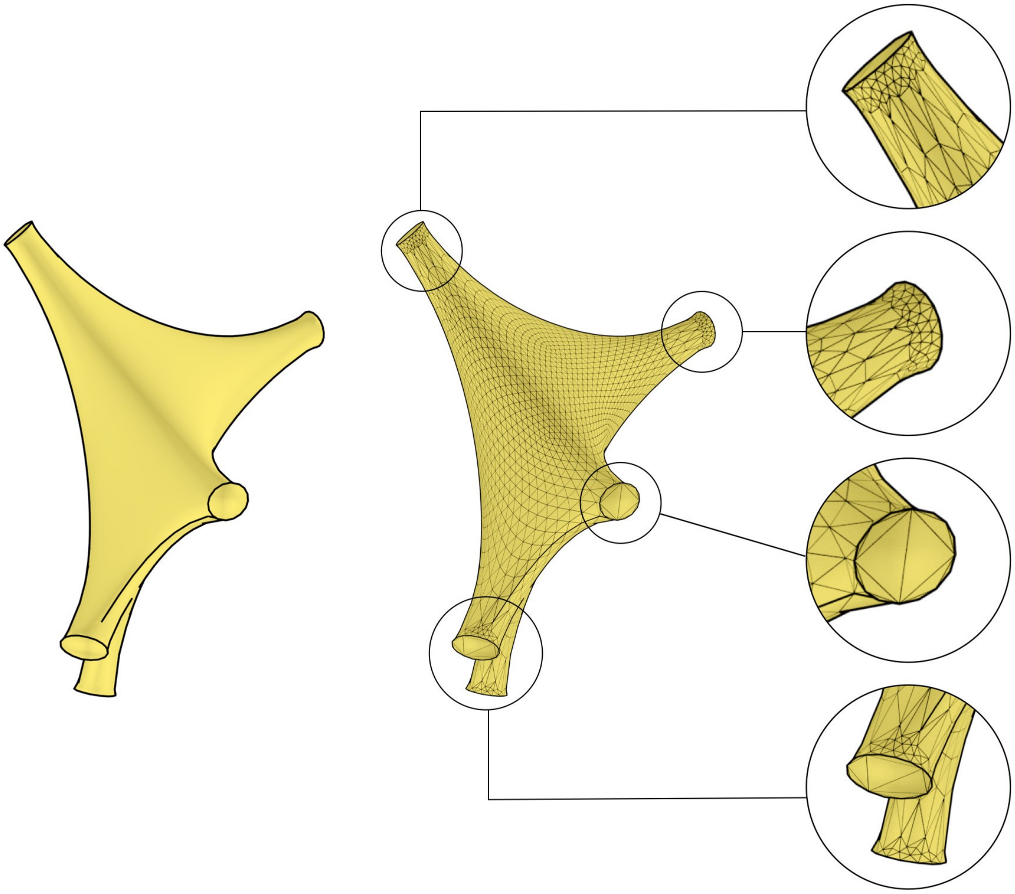

Figure S12. The somatic profile of the astrocyte as reconstructed with the Soft Body method in the soma reconstruction toolbox in NeuroMorphoVis (?). The vertices are more condensed towards the arbors.

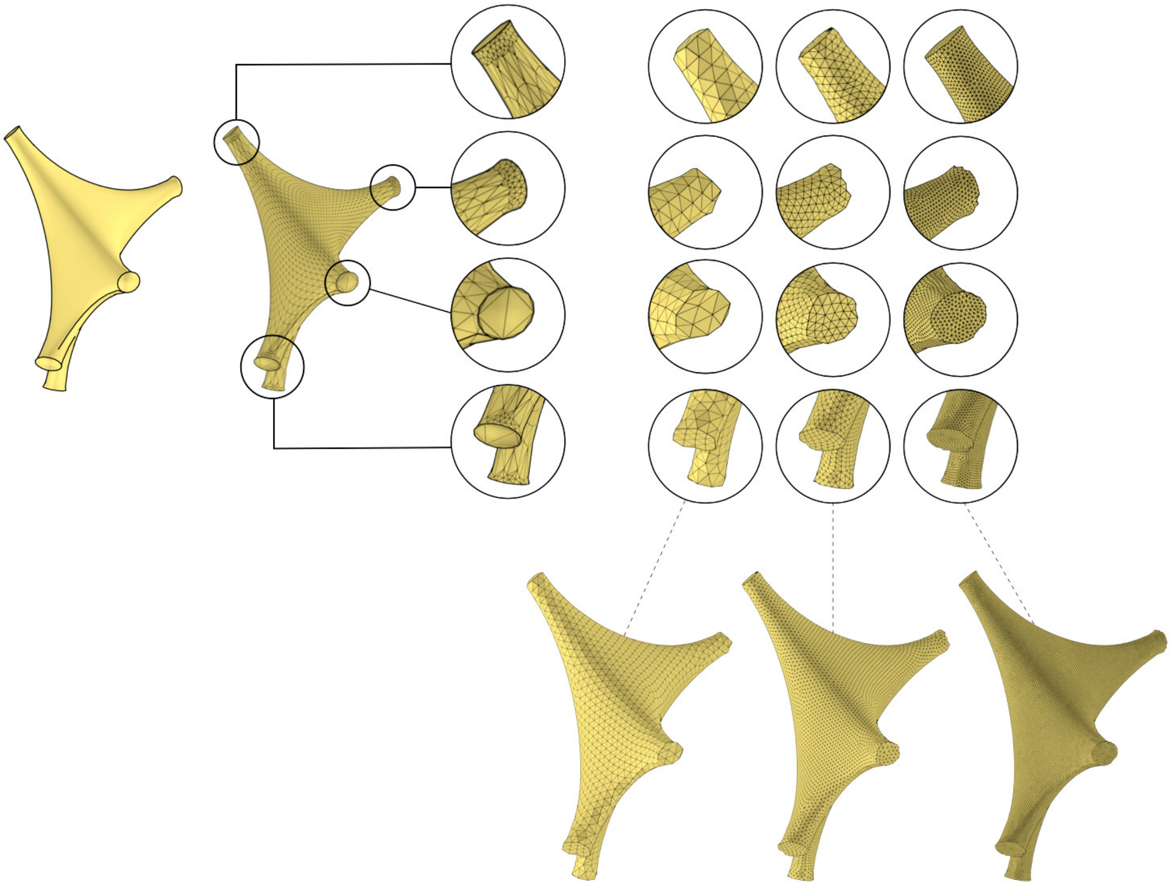

Figure S13. The point cloud (or vertices) of the initial surface mesh obtained from soft body simulation in NeuroMorphoVis is not uniformly distributed (left). We therefore use a particle system remesher to create uniformly tessellated surface mesh to be used for skinning the somatic surface with metaballs. The resulting mesh is subdivided with one, two and three subdivision levels.

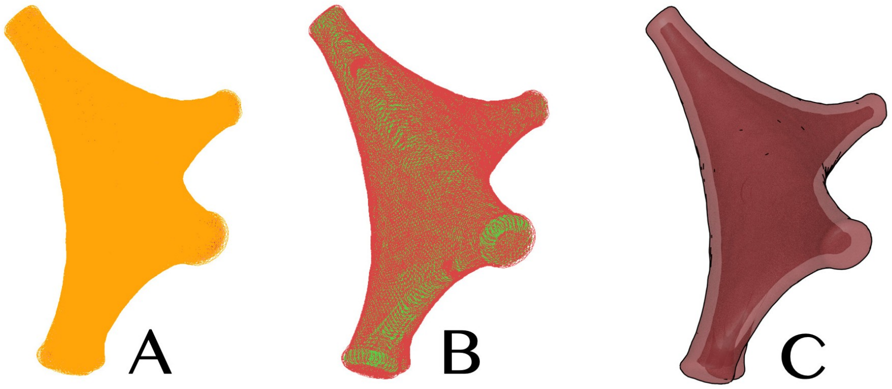

Figure S14. For each vertex in the uniformly-sampled mesh obtained in Figure S13, a metaball object will be created. (A) The final meta-soma after converting all the vertices of the input mesh into metaballs. (B) The meta-soma in the edit mode showing the influence of every metaball in the base object. (C) The reconstructed mesh is composed of two surfaces or partitions.

1.7 Endfeet reconstruction

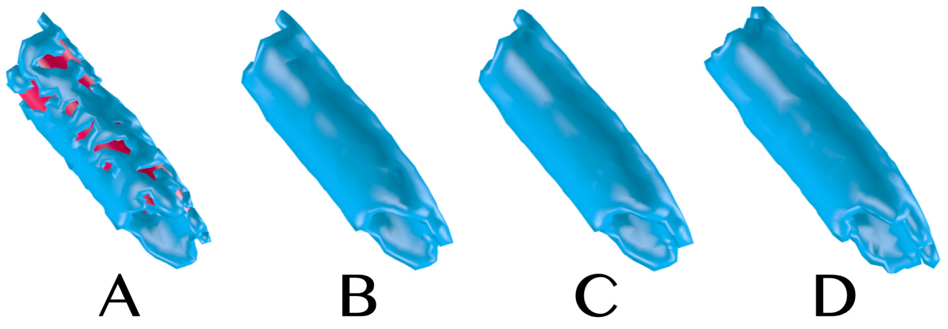

Figure S15. A given endfoot patch cannot be processed directly to build a corresponding meta-object due to the limited tessellation of the patch with respect to the endfoot thickness. If this patch is used to reconstruct the meta-object, it will merely create a group of fragmented mesh partitions as shown in (A). Using the subdivision iterator improves the reconstructed surface. The endfoot patch is subdivided with levels one (B), two (C) and three (D).

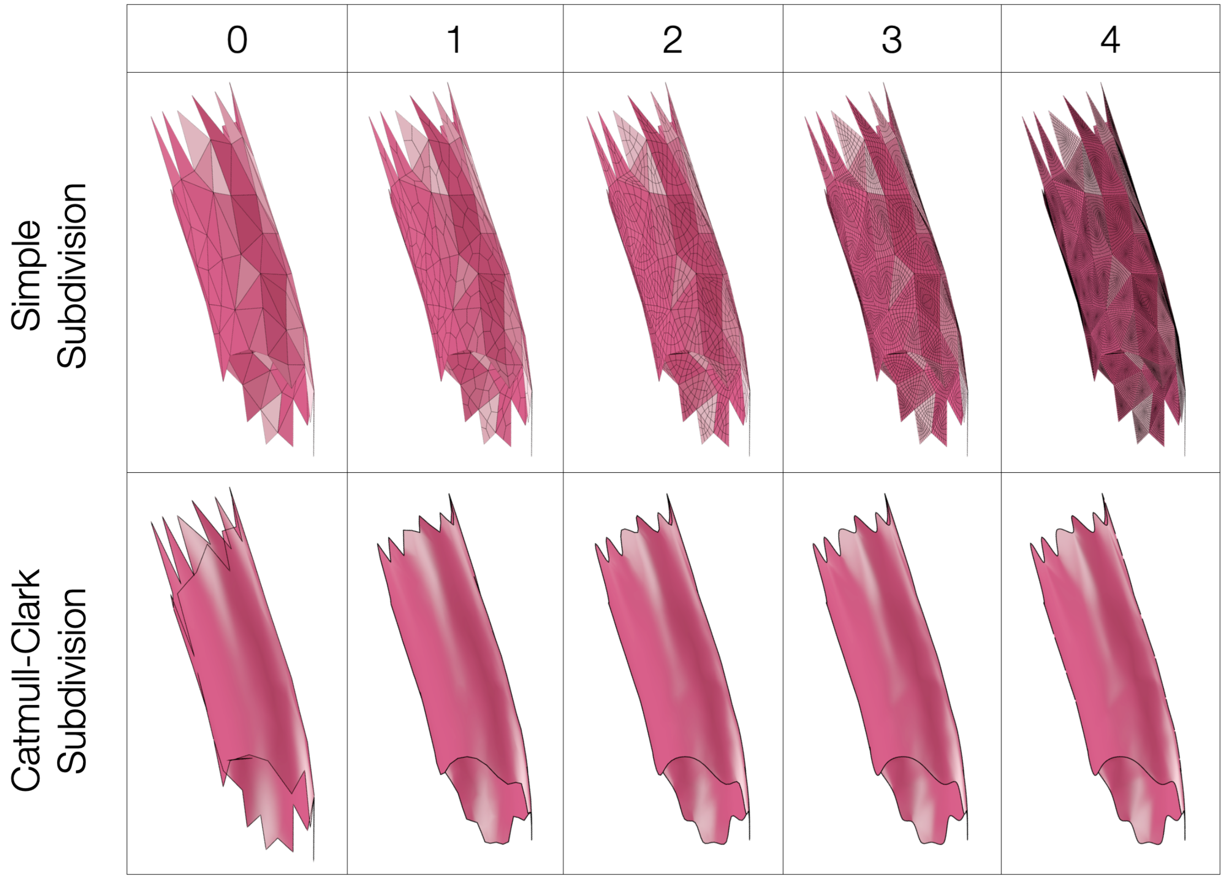

Figure S16. A comparison between using simple and Catmull-Clark subdivision in Blender to subdivide the endfeet patch with different subdivision levels.

1.8 Reconstructed meshes

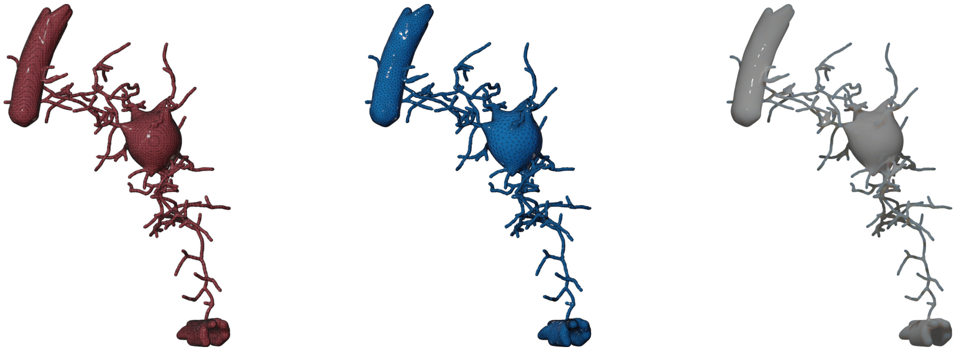

Figure S17. Wireframe renderings for a mesh created with our metaball skinning implementation (in red), an optimized one (in blue) and a high quality rendering of the optimized mesh for the astrocytic morphology with GID=2.

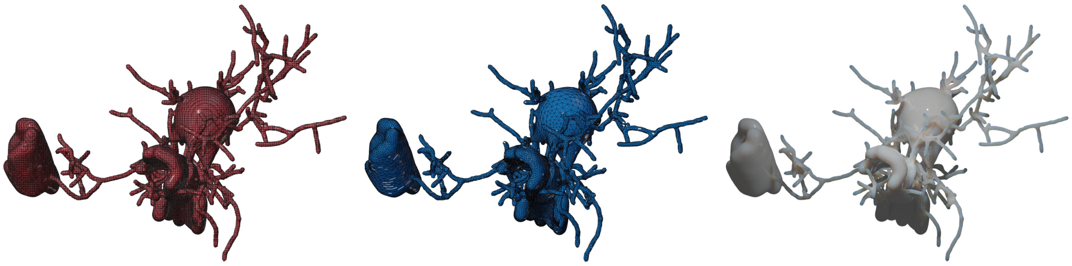

Figure S18. Wireframe renderings for a mesh created with our metaball skinning implementation (in red), an optimized one (in blue) and a high quality rendering of the optimized mesh for the astrocytic morphology with GID=3.

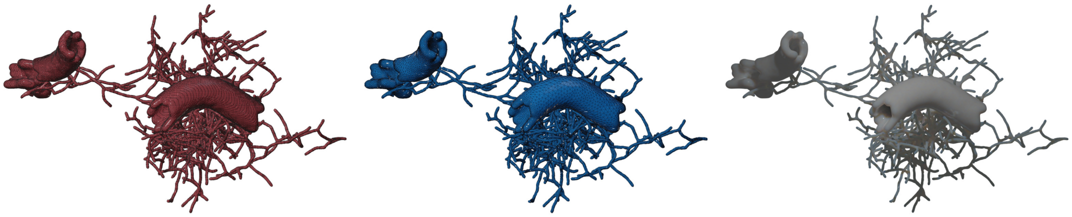

Figure S19. Wireframe renderings for a mesh created with our metaball skinning implementation (in red), an optimized one (in blue) and a high quality rendering of the optimized mesh for the astrocytic morphology with GID=5.

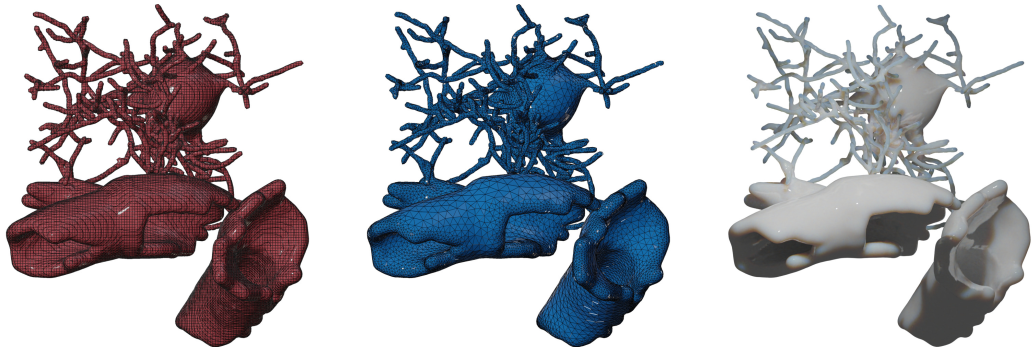

Figure S20. Wireframe renderings for meshes created with our metaball skinning implementation (in red), the optimized ones (in blue) and a high quality rendering of the optimized mesh for astrocytic morphology with GID=10.

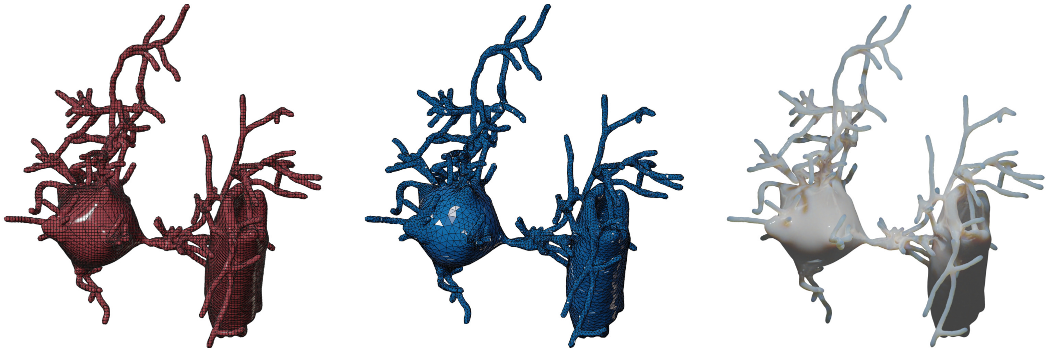

Figure S21. Wireframe renderings for meshes created with our metaball skinning implementation (in red), the optimized ones (in blue) and a high quality rendering of the optimized mesh for astrocytic morphology with GID=15.

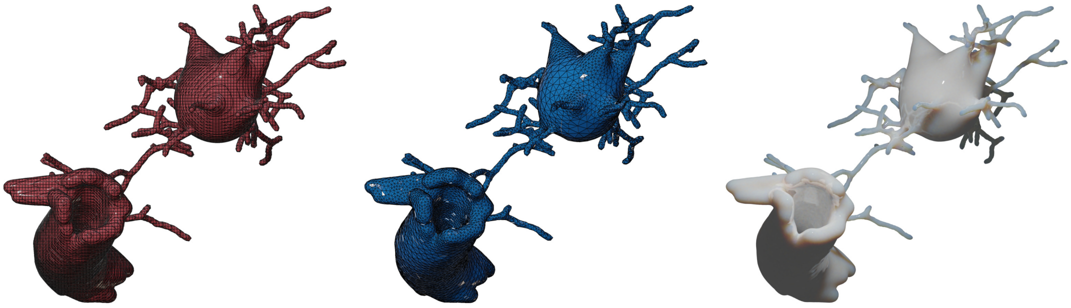

Figure S22. Wireframe renderings for meshes created with our metaball skinning implementation (in red), the optimized ones (in blue) and a high quality rendering of the optimized mesh for astrocytic morphology with GID=18.

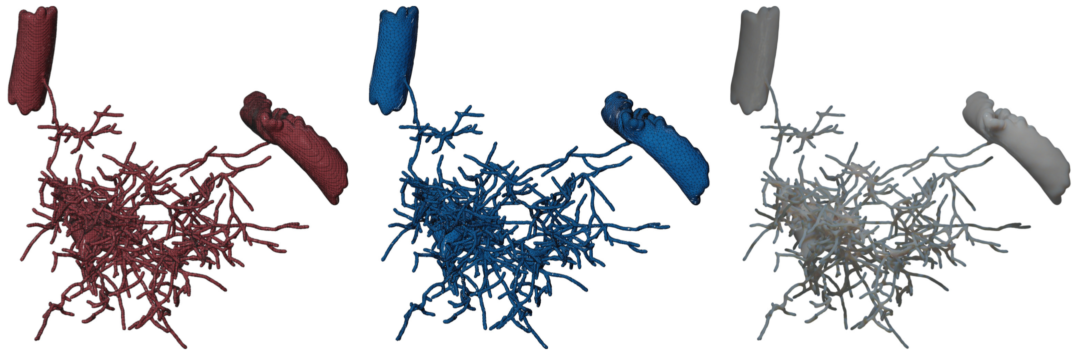

Figure S23. Wireframe renderings for meshes created with our metaball skinning implementation (in red), the optimized ones (in blue) and a high quality rendering of the optimized mesh for astrocytic morphology with GID=20.

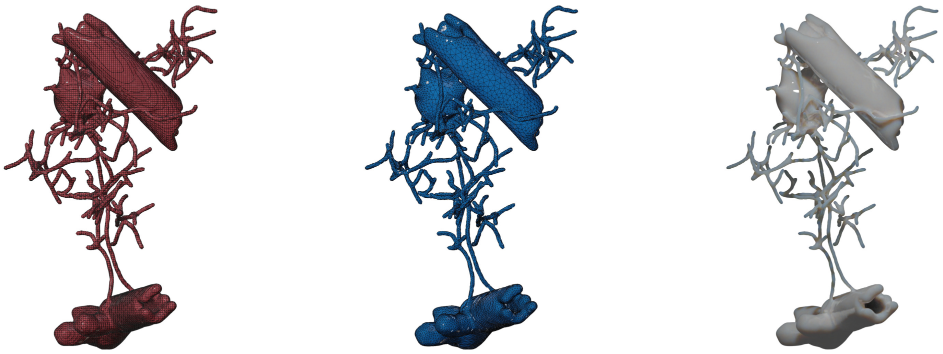

Figure S24. Wireframe renderings for meshes created with our metaball skinning implementation (in red), the optimized ones (in blue) and a high quality rendering of the optimized mesh for astrocytic morphology with GID=25.

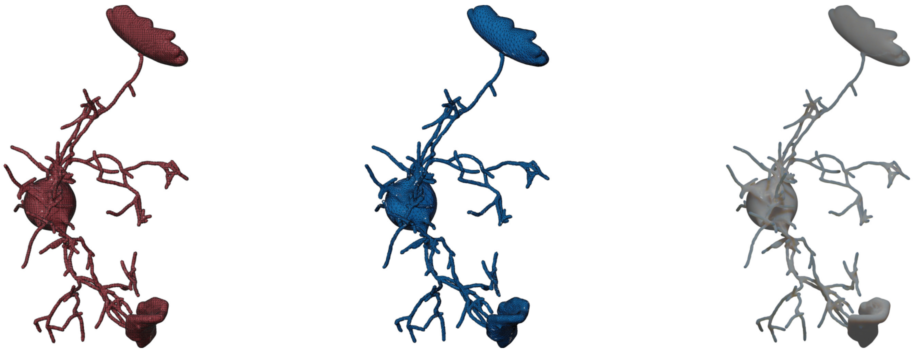

Figure S25. Wireframe renderings for meshes created with our metaball skinning implementation (in red), the optimized ones (in blue) and a high quality rendering of the optimized mesh for astrocytic morphology with GID=30.

1.9 Qualitative measures

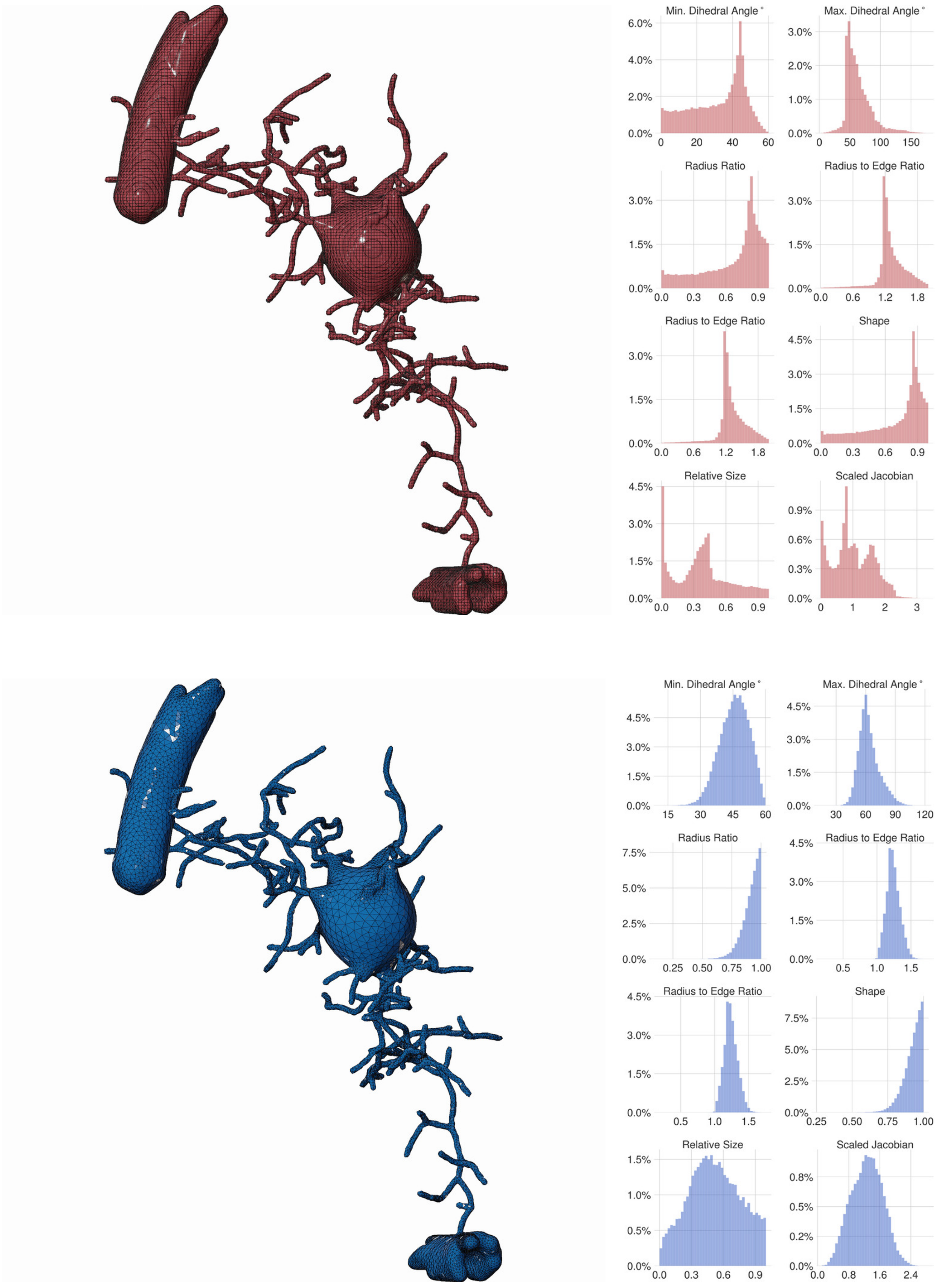

Figure S26. Analytical comparison between (i) the astrocytic mesh created with our metaball skinning implementation (in red), and (ii) the optimized mesh (in blue) for the astrocytic morphology with GID=2 in the NGV circuit.

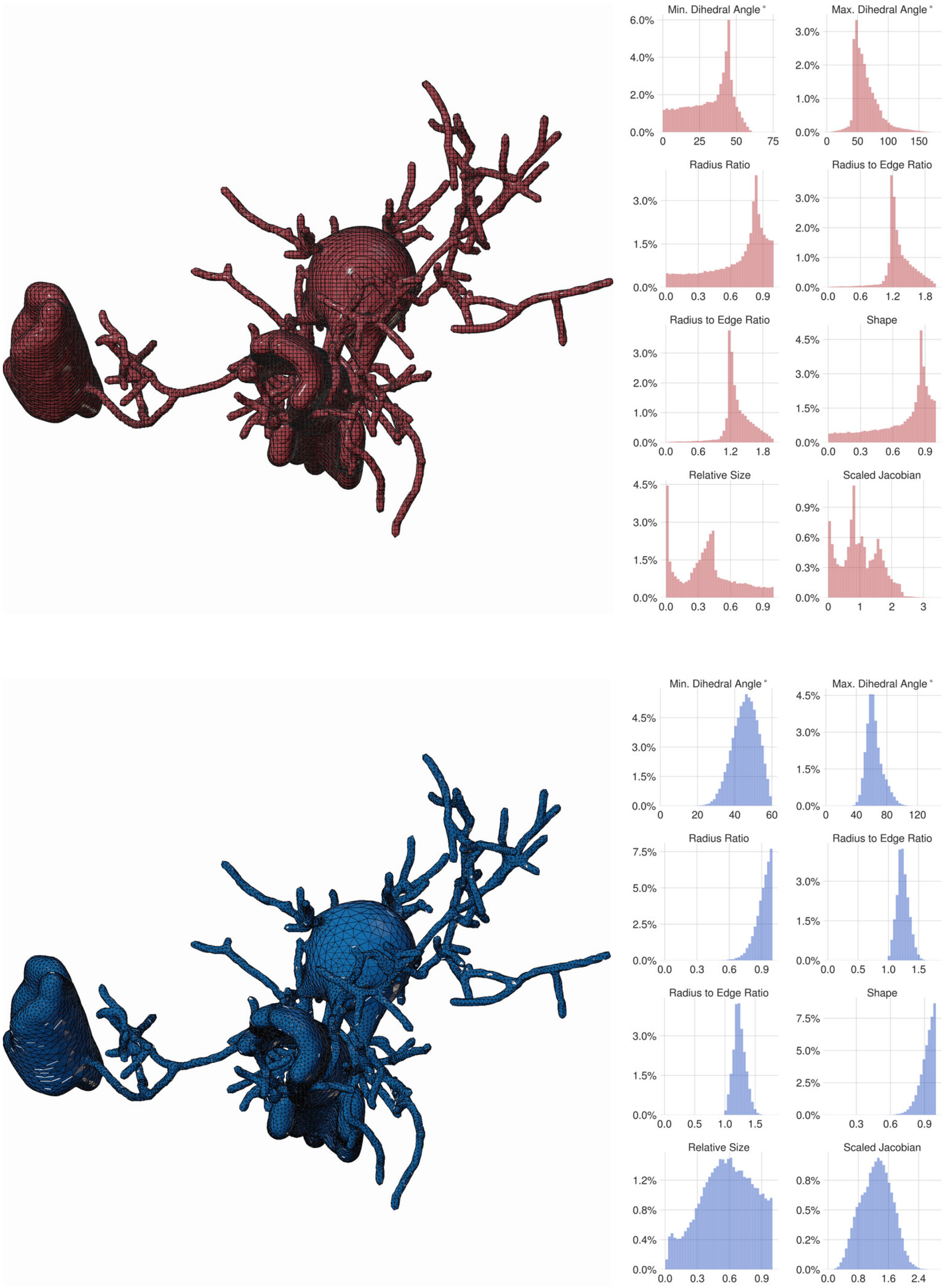

Figure S27. Analytical comparison between (i) the astrocytic mesh created with our metaball skinning implementation (in red), and (ii) the optimized mesh (in blue) for the astrocytic morphology with GID=3 in the NGV circuit.

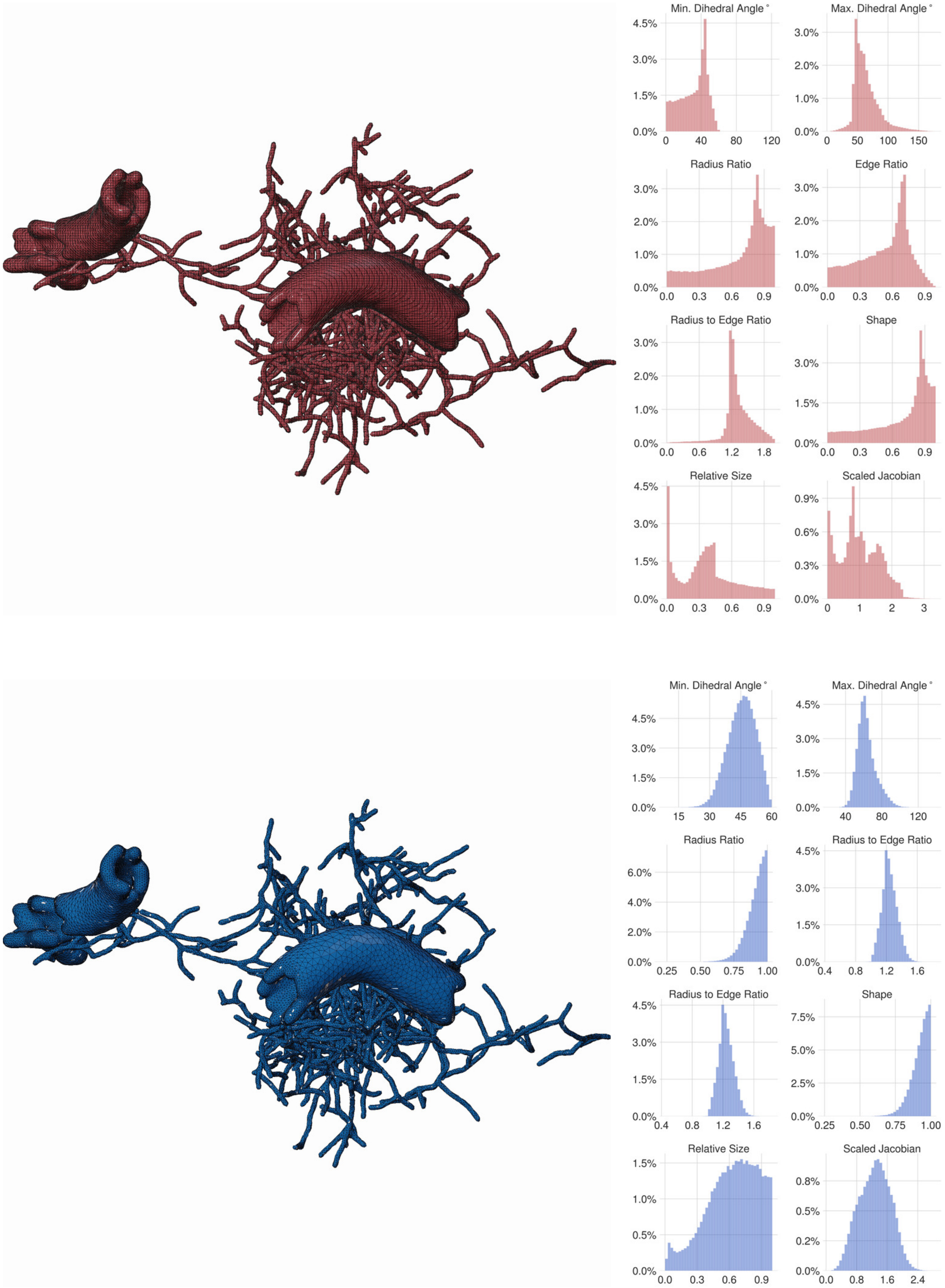

Figure S28. Analytical comparison between (i) the astrocytic mesh created with our metaball skinning implementation (in red), and (ii) the optimized mesh (in blue) for the astrocytic morphology with GID=5 in the NGV circuit.

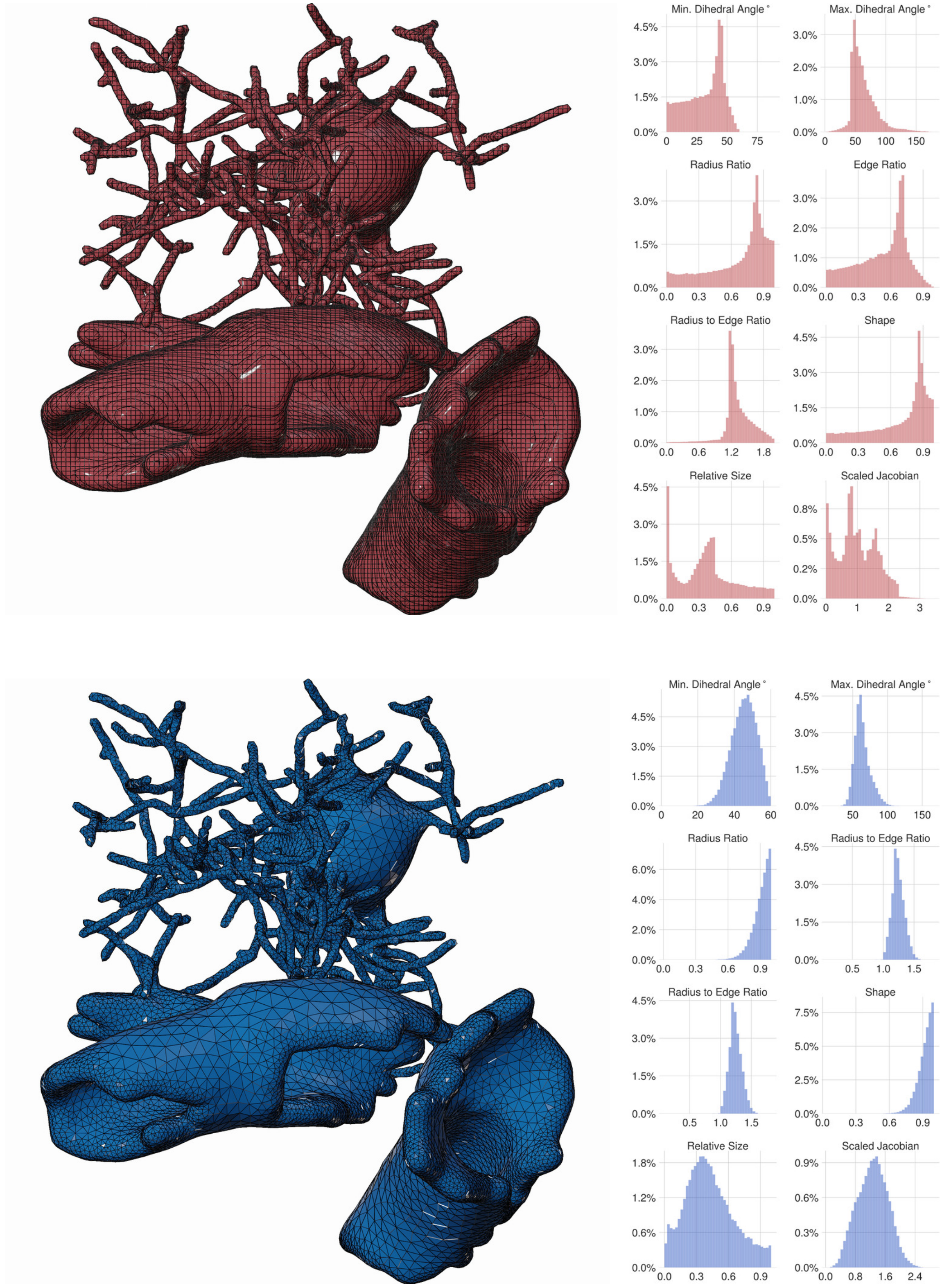

Figure S29. Analytical comparison between (i) the astrocytic mesh created with our metaball skinning implementation (in red), and (ii) the optimized mesh (in blue) for the astrocytic morphology with GID=10 in the NGV circuit.

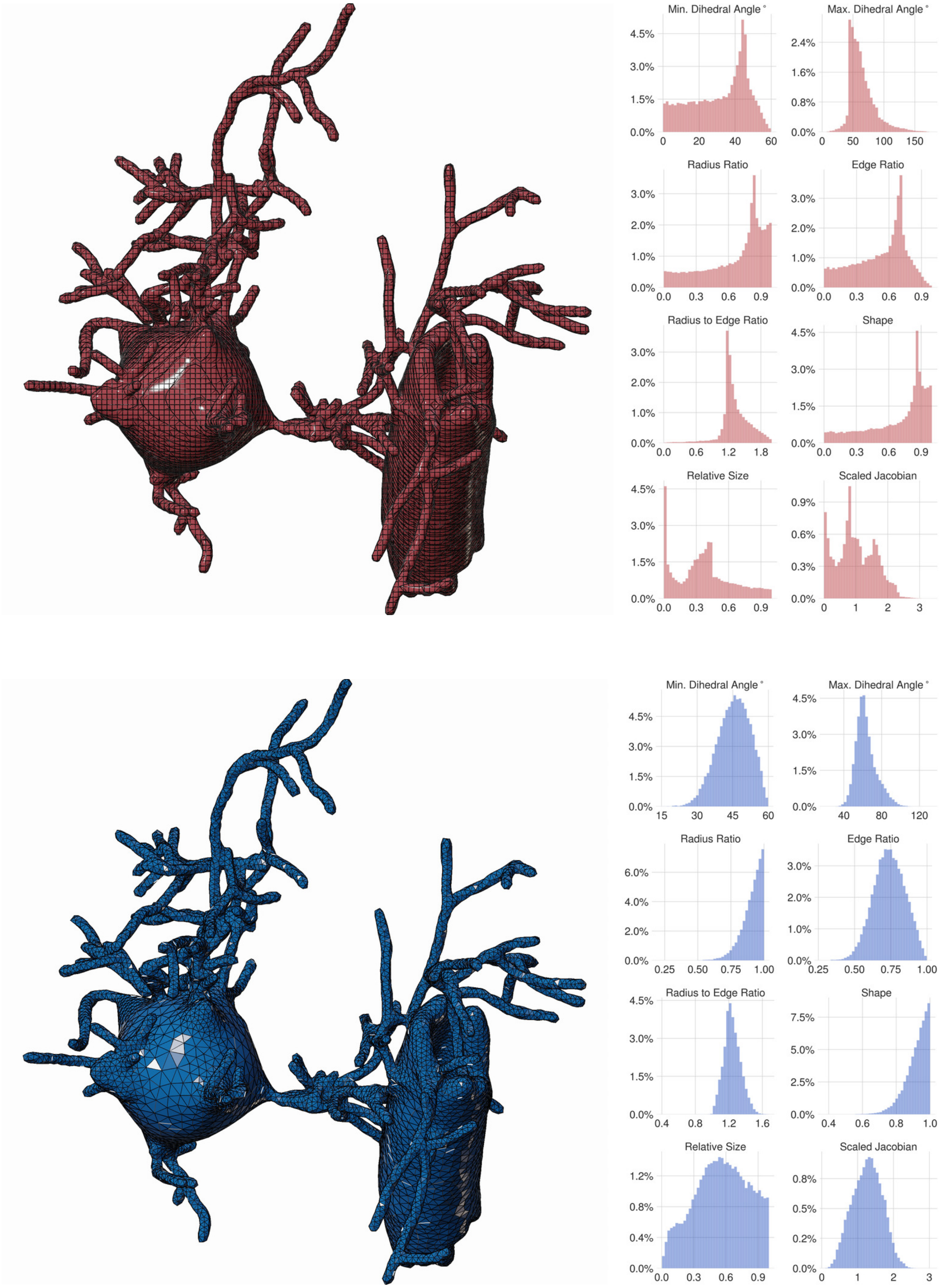

Figure S30. Analytical comparison between (i) the astrocytic mesh created with our metaball skinning implementation (in red), and (ii) the optimized mesh (in blue) for the astrocytic morphology with GID=15 in the NGV circuit.

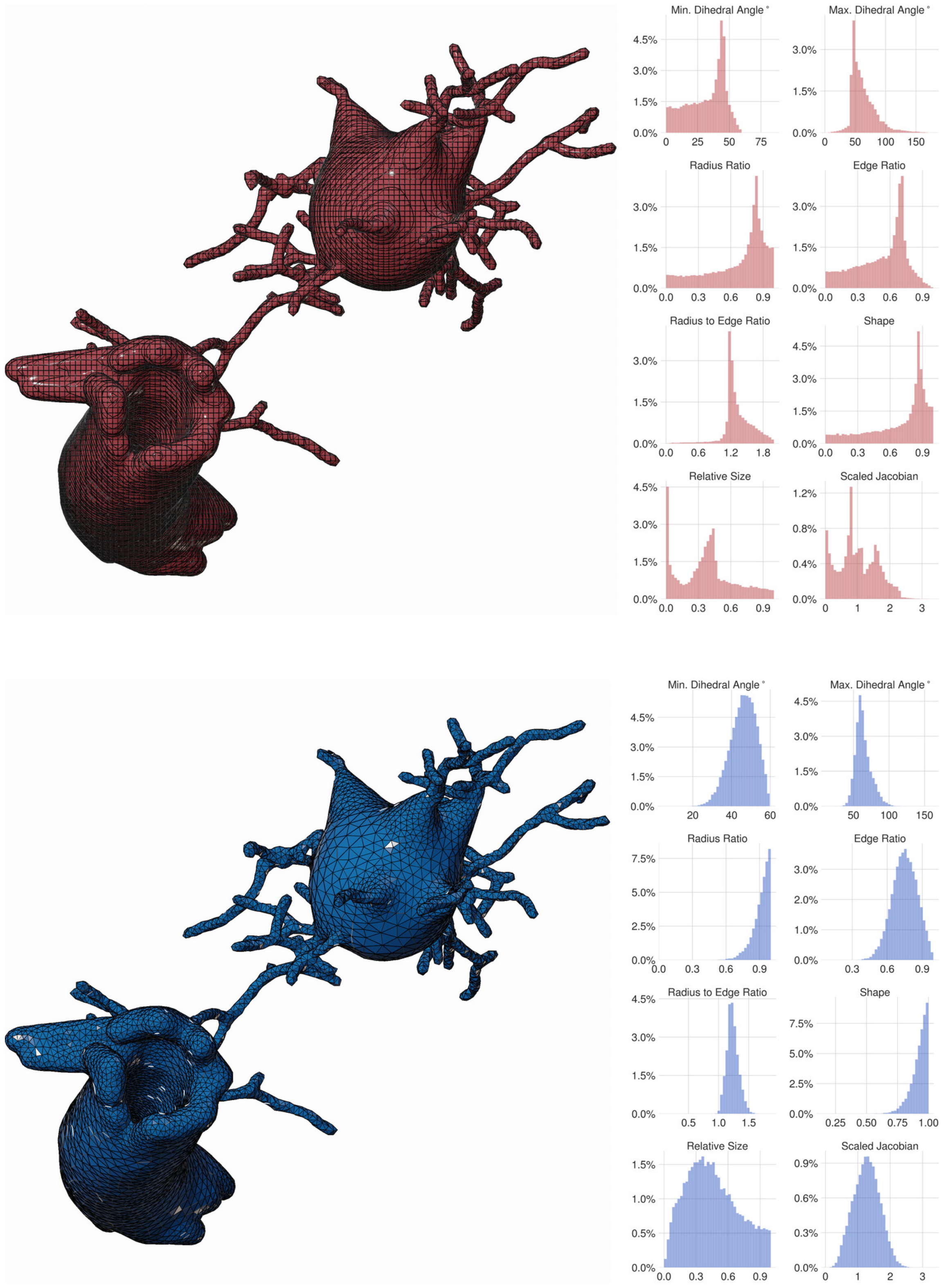

Figure S31. Analytical comparison between (i) the astrocytic mesh created with our metaball skinning implementation (in red), and (ii) the optimized mesh (in blue) for the astrocytic morphology with GID=18 in the NGV circuit.

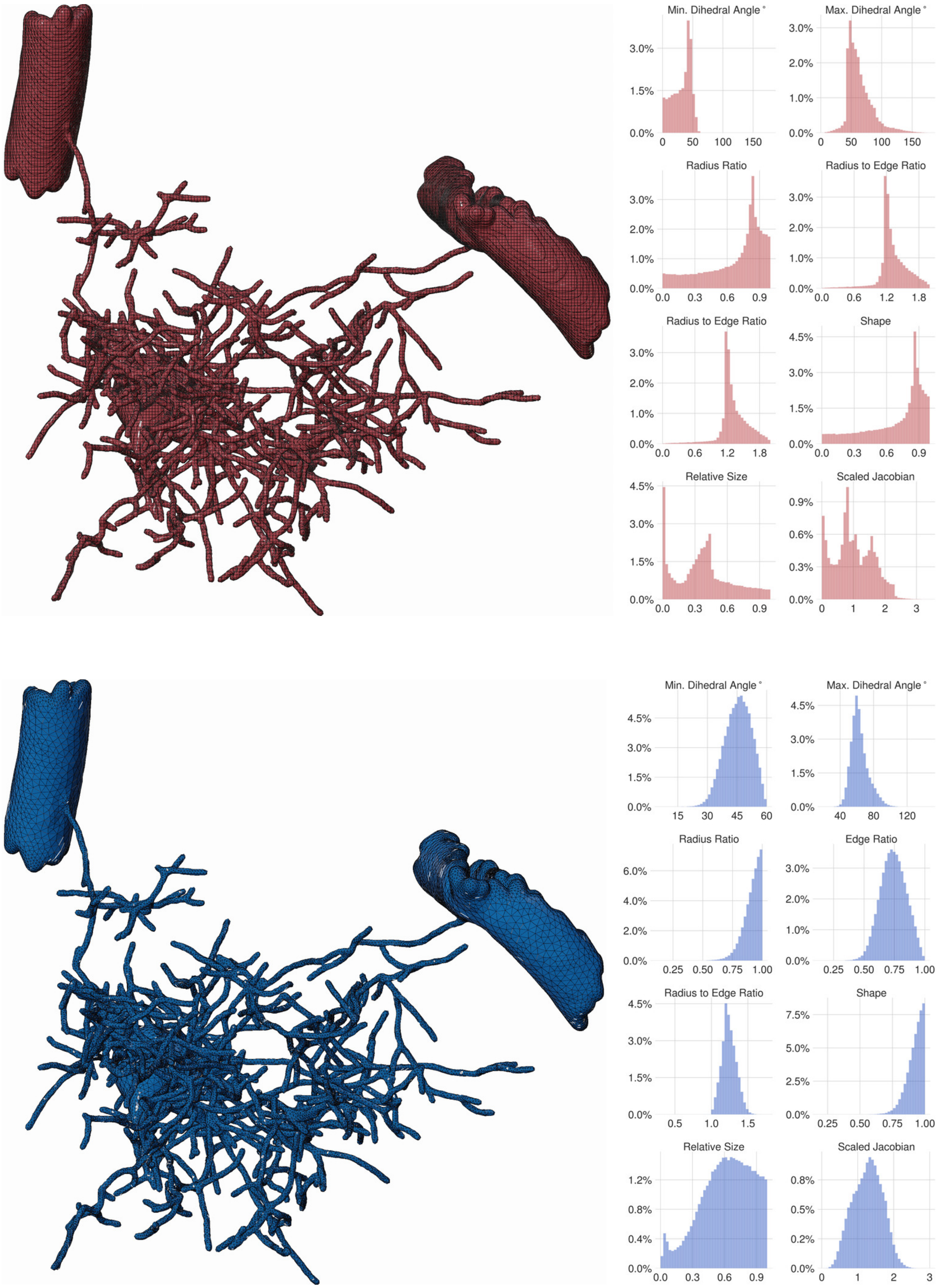

Figure S32. Analytical comparison between (i) the astrocytic mesh created with our metaball skinning implementation (in red), and (ii) the optimized mesh (in blue) for the astrocytic morphology with GID=20 in the NGV circuit.

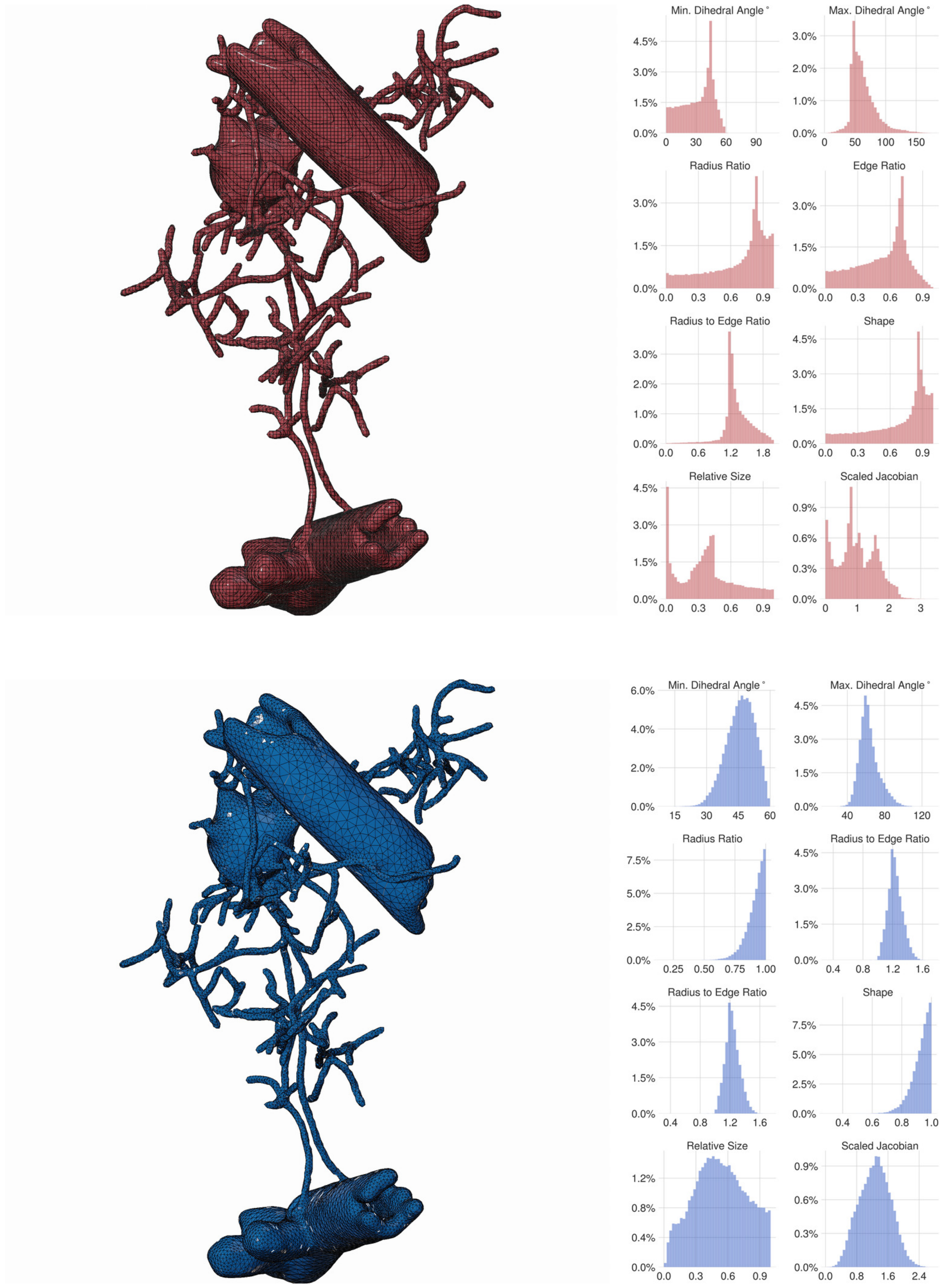

Figure S33. Analytical comparison between (i) the astrocytic mesh created with our metaball skinning implementation (in red), and (ii) the optimized mesh (in blue) for the astrocytic morphology with GID=25 in the NGV circuit.

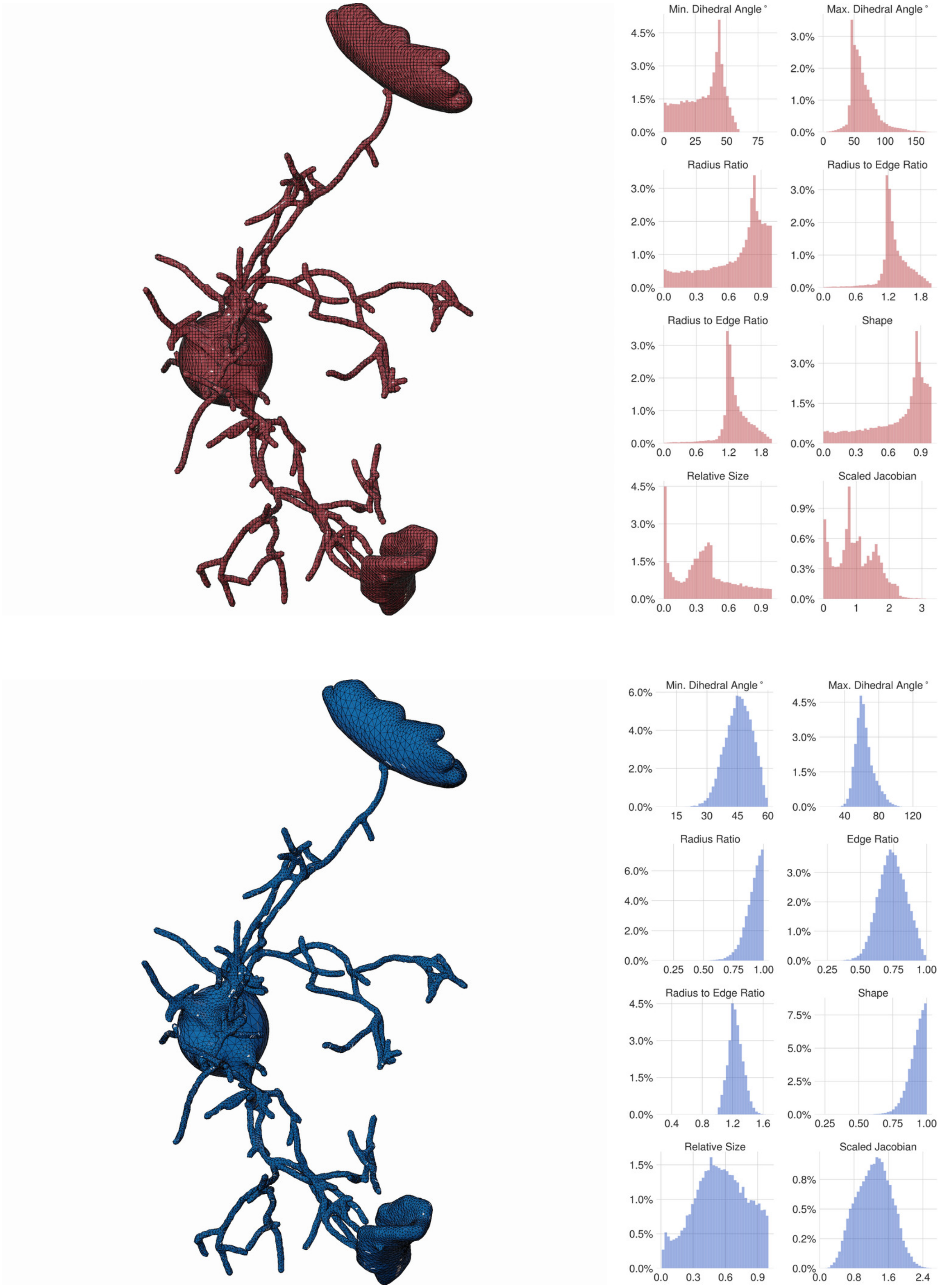

Figure S34. Analytical comparison between (i) the astrocytic mesh created with our metaball skinning implementation (in red), and (ii) the optimized mesh (in blue) for the astrocytic morphology with GID=30 in the NGV circuit.

1.10 Visual analytics

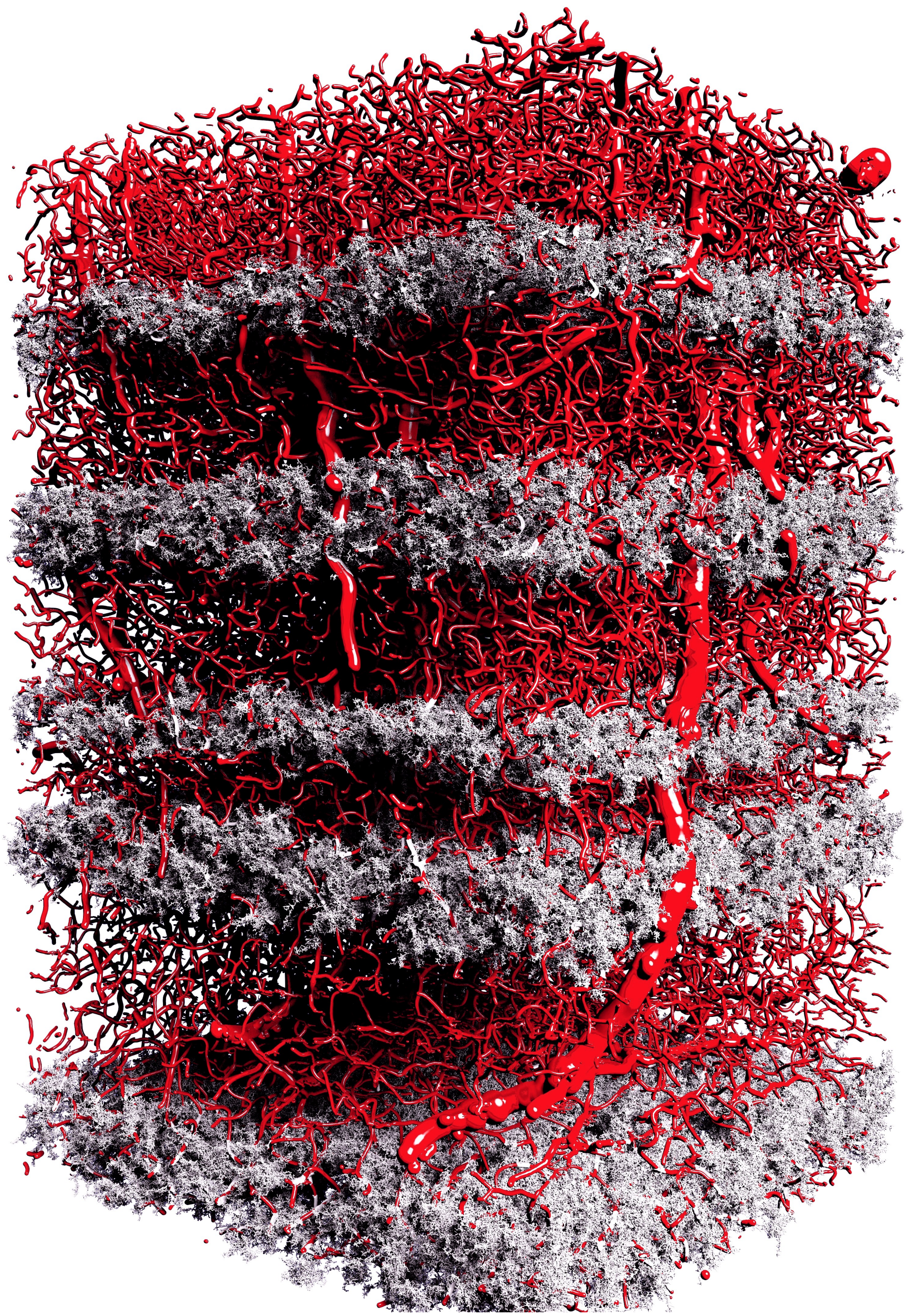

Figure S35. A high-quality rendering of the vasculature mesh shown in Figure S4 combined with the 5000 astrocytic meshes that were generated with our pipeline. The total size of the astrocytic meshes is ∼20 GBytes. The image is rendered with the OSPRay rendering engine that is integrated in Brayns. This visual is used to verify the correct placement of the astrocyte in the NGV circuit.

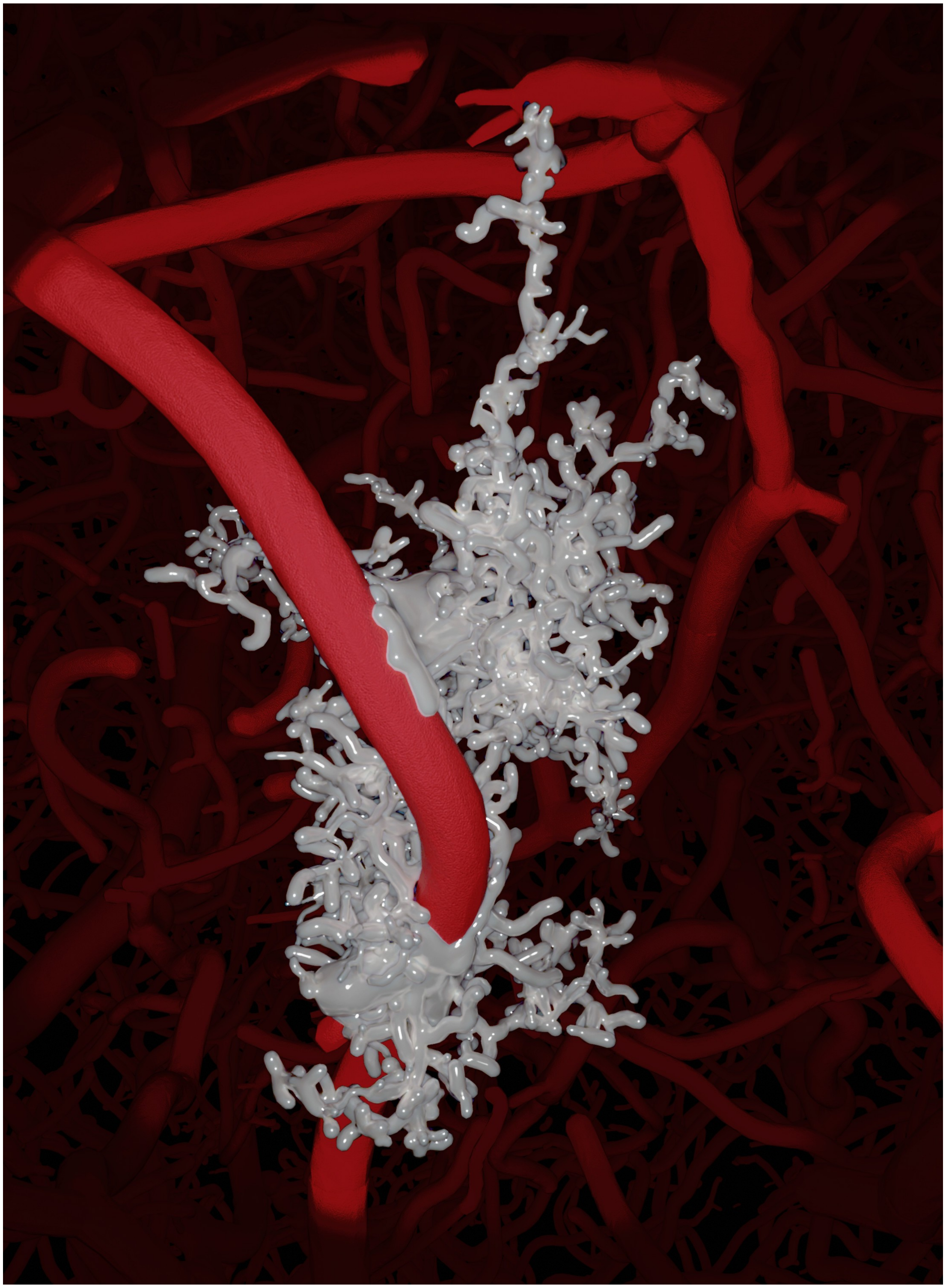

Figure S36. The generated meshes are used to verify the structural aspects of the NGV circuit including the astrocytic cell placement and their connectivity to the vasculature. The image is rendered in Blender using Cycles.

1.11 Visualizing reaction-diffusion simulation reports

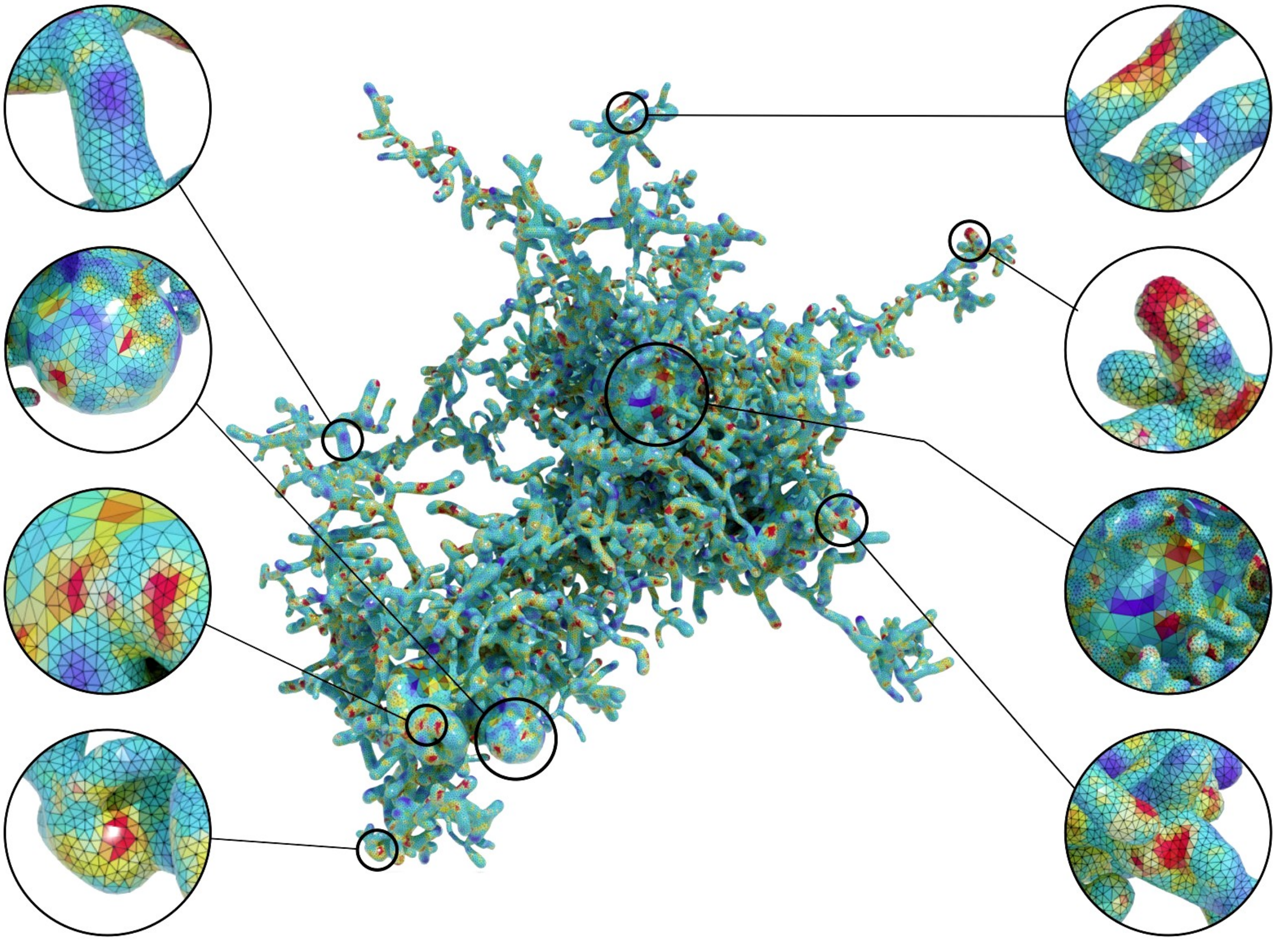

Figure S37. A tetrahedral mesh of an exemplar astrocyte showing a randomly-generated simulation report to mimic the variations of Ca^{+2} concentrations across the astrocytic surface. This tetrahedral mesh is created with QuarTet using an input surface mesh reconstructed with our metaball skinning add-on.

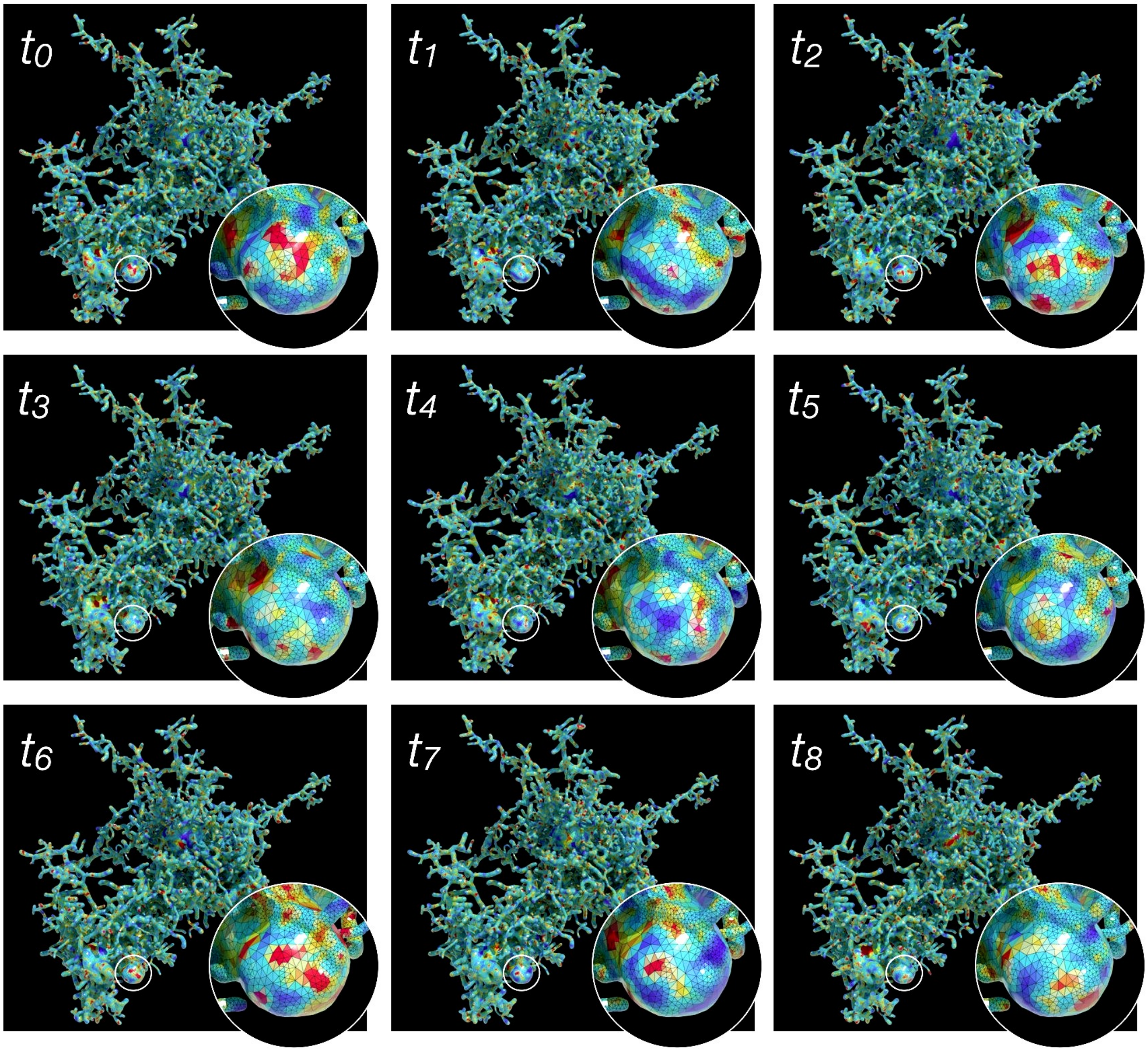

Figure S38. Visualizing a time series simulation.

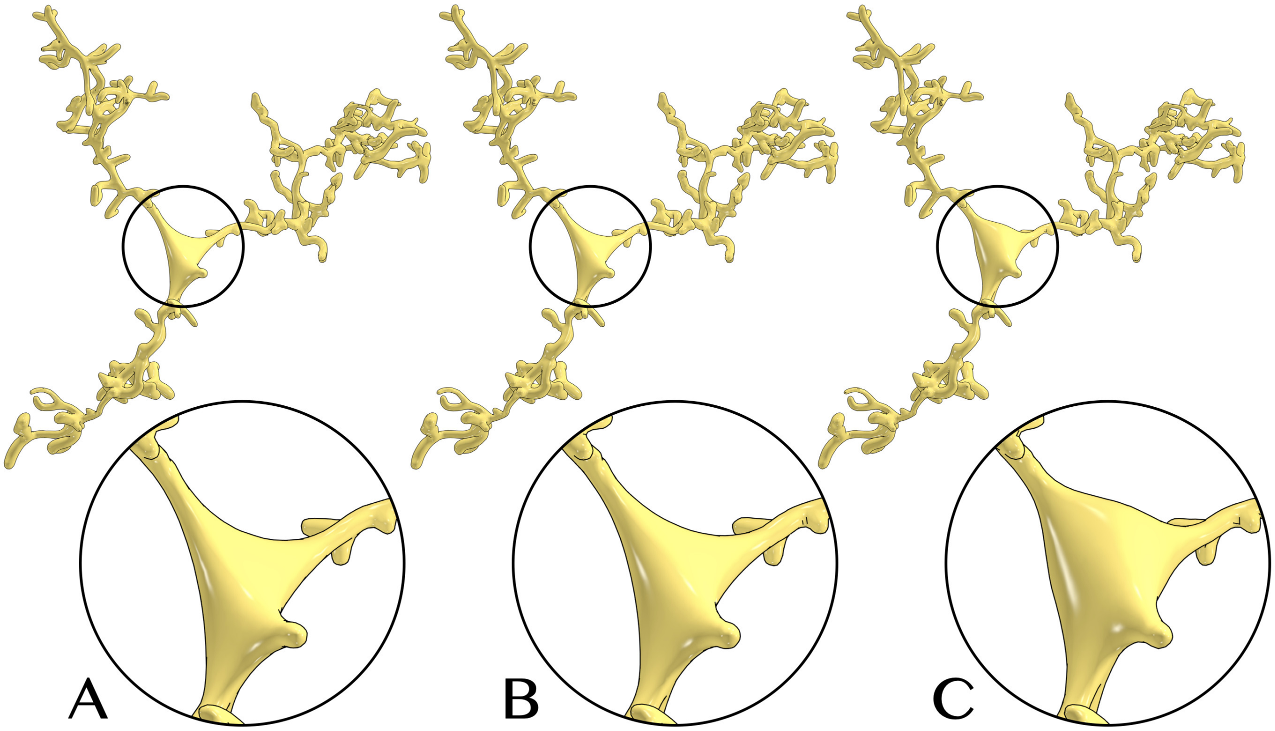

Figure S39. The hybrid approach allows the creation of various somatic profiles by tweaking the soft body parameters, including stiffness and number of simulation steps.

1.12 General remarks

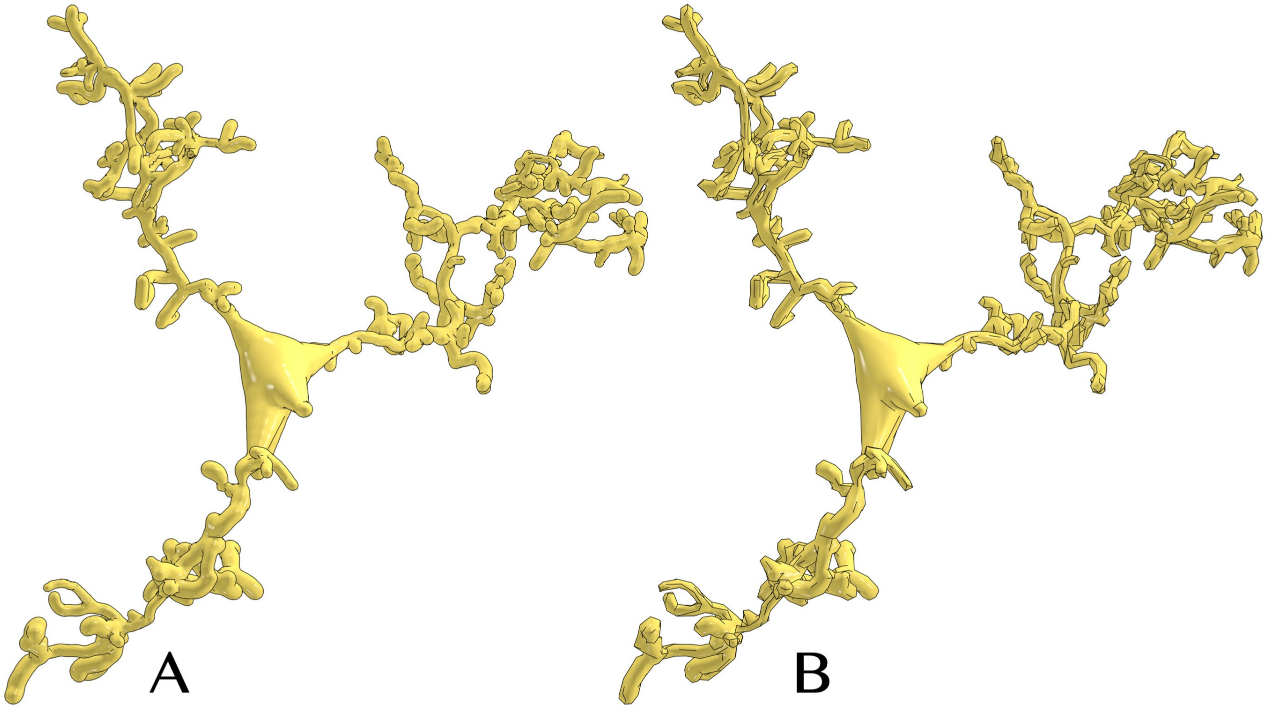

Figure S40. A visual comparison between two astrocyte meshes with and without decimation. The mesh in (A) is not decimated, while that in (B) is decimated with a decimation ratio of 0.1.

2 Running the pipeline

The pipeline can be called from a simple CLI and also can be configured with a bash configuration file. The pipeline has been tested on Ubuntu, RedHat and macOSX.

2.1 Command line interface

```
usage: run.py   [-h]
                [--blender-executable BLENDER_EXECUTABLE]
                [--output-directory OUTPUT_DIRECTORY] [--mesh-type MESH_TYPE]
                [--execution EXECUTION] [--number-cores NUMBER_CORES]
                [--circuit-path CIRCUIT_PATH] [--soma-style SOMA_STYLE]
                [--gids-file GIDS_FILE] [--gids-range GIDS_RANGE] [--export-obj]
                [--export-blend] [--create-optimized]
                [--ultra-clean-mesh-executable ULTRA_CLEAN_MESH_EXECUTABLE]
                [--decimation-factor DECIMATION_FACTOR]

Generating astrocytes

optional arguments:
-h, --help            show this help message and exit
--blender-executable BLENDER_EXECUTABLE
     Blender executable
--output-directory OUTPUT_DIRECTORY
     Output directory where the generated astrocyte will be written
--mesh-type MESH_TYPE
     The type of the resulting meshes, [simulation], [visualization] or [both]
--execution EXECUTION
     Execution mode, serial or parallel
--number-cores NUMBER_CORES
     Number of cores for parallel processing
--circuit-path CIRCUIT_PATH
     The path to the NGV circuit
--soma-style SOMA_STYLE
     The style of the soma
--gids-file GIDS_FILE
     The GIDs of the astrocytes
--gids-range GIDS_RANGE
     A range of GIDs
--export-obj
     Export the result into an .OBJ file
--export-blend
     Export the result into an .BLEND file
--create-optimized
     Create the optimized mesh
--ultra-clean-mesh-executable ULTRA_CLEAN_MESH_EXECUTABLE
     ultraCleanMesh executable
--decimation-factor DECIMATION_FACTOR
     Decimation factor, between 1.0 and 0.01
```

2.2 Configurable shell script

```
#!/usr/bin/env bash
####################################################################################################
# Copyright (c) 2020, EPFL / Blue Brain Project
#               Marwan Abdellah <marwan.abdellah@epfl.ch>
#
# This file is part of NeuroMorphoVis <https://github.com/BlueBrain/NeuroMorphoVis>
#
# This program is free software: you can redistribute it and/or modify it under the terms of
# the GNU General Public License as published by the Free Software Foundation, version 3 of
# the License.
#
# This Blender-based tool is distributed in the hope that it will be useful, but WITHOUT ANY
# WARRANTY; without even the implied warranty of MERCHANTABILITY or FITNESS FOR A PARTICULAR
# PURPOSE.  See the GNU General Public License for more details.
#
# You should have received a copy of the GNU General Public License along with this program.
# If not, see <http://www.gnu.org/licenses/>.
####################################################################################################

# Blender executable, version 2.8x or 2.9x
BLENDER=PLEASE_SPECIFY_BLENDER_EXECUTABLE

# Output directory, where the results will be generated
OUTPUT_DIRECTORY=PLEASE_PROVIDE_BLENDER_EXECUTABLE

# NGV Circuit
CIRCUIT=PLEASE_PROVIDE_A_MULTI_POPULATED_CIRCUIT

# Soma style, [metaball] or [softbody]
SOMA_STYLE='metaball'

# A list of GIDs, if this is defined the GIDS_FILE is ignored, and if set to '0' the file is used
GIDS_RANGE=PLEASE_SET_A_RANGE_OF_GIDS

# GIDs file (a file contains a list of GIDs of the astrocytes to be reconstructed separated by space)
GIDS_FILE=PLEASE_PROVIDE_A_LIST_OF_GIDS

# Meshes type, use [simulation] or [visualization] or [both]
MESH_TYPE='visualization'

# Create optimized meshes, [yes] or [no]
CREATE_OPTIMIZED='yes'

# ultraCleanMesh executable (for optimization and re-tesselation)
# ULTRA_CLEAN_MESH_EXECUTABLE='ultraCleanMesh'

# Execution, [serial] or [parallel]
EXECUTION='parallel'

# Number of cores if parallel processing is set in the EXECUTION option
NUMBER_CORES=PLEASE_SET_THE_NUMBER_OF_CORES

# Decimation factor (range: 0.5 - 0.01) to reduce the number of polygons in the mesh that is
# generated for the visualization purposes.
DECIMATION_FACTOR=PLEASE_SET_THE_DECIMATION_FACTOR

# Export the final mesh in a .OBJ file, [yes] or [no]
EXPORT_OBJ='yes'

# Export the final mesh in a .BLEND file, [yes] or [no]
```

```
EXPORT_BLEND='yes'

#################################################################################################
BOOL_ARGS=''
if [ "$EXPORT_OBJ" == 'yes' ];
then BOOL_ARGS+=' --export-obj '; fi
if [ "$EXPORT_BLEND" == "yes" ];
then BOOL_ARGS+=' --export-blend '; fi
if [ "$CREATE_OPTIMIZED" == "yes" ];
then BOOL_ARGS+=' --create-optimized '; fi

#################################################################################################
$PWD/../../../../../python/bin/python3.7m run.py                                                  \
--blender-executable=$BLENDER                                                                     \
--gids-file=$GIDS_FILE                                                                            \
--gids-range=$GIDS_RANGE                                                                          \
--soma-style=$SOMA_STYLE                                                                          \
--execution=$EXECUTION                                                                            \
--circuit-path=$CIRCUIT                                                                           \
--mesh-type=$MESH_TYPE                                                                            \
--ultra-clean-mesh-executable=$ULTRA_CLEAN_MESH_EXECUTABLE                                        \
--decimation-factor=$DECIMATION_FACTOR                                                            \
--output-directory=$OUTPUT_DIRECTORY                                                              \
--number-cores=$NUMBER_CORES                                                                      \
$BOOL_ARGS
#################################################################################################
```

3 Software repositories

1. Blender: https://www.blender.org/ & https://github.com/blender/blender
2. NeuroMorphoVis: https://github.com/BlueBrain/NeuroMorphoVis
3. GAMer: http://fetk.org/codes/gamer
4. MeshFix: https://github.com/MarcoAttene/MeshFix-V2.1
5. QuarTet: https://github.com/crawforddoran/quartet
